# Development of a nano-emulsion based multivalent protein subunit vaccine against *Pseudomonas aeruginosa*

**DOI:** 10.3389/fimmu.2024.1372349

**Published:** 2024-04-18

**Authors:** Debaki R. Howlader, Rahul Shubhra Mandal, Ti Lu, Suhrid Maiti, Zackary K. Dietz, Sayan Das, Sean K. Whittier, Aaron C. Nagel, Satabdi Biswas, David J. Varisco, Francesca M. Gardner, Robert K. Ernst, William D. Picking, Wendy L. Picking

**Affiliations:** ^1^ Department of Veterinary Pathobiology, Center for Veterinary Medicine, University of Missouri, Columbia, MO, United States; ^2^ Bond Life Science Center, University of Missouri, Columbia, MO, United States; ^3^ Department of Pharmaceutical Chemistry, University of Kansas, Lawrence, KS, United States; ^4^ Perelman School of Medicine, University of Pennsylvania, Philadelphia, PA, United States; ^5^ Department of Microbial Pathogenesis, University of Maryland, Baltimore, MD, United States; ^6^ Hafion, Inc., Lawrence, KS, United States

**Keywords:** pseudomonas, T3SS vaccine, L-PaF, nano-emulsion, nanoparticle vaccine, RNA seq, IL-17A

## Abstract

*Pseudomonas aeruginosa* (Pa) is an opportunistic bacterial pathogen responsible for severe hospital acquired infections in immunocompromised and elderly individuals. Emergence of increasingly drug resistant strains and the absence of a broad-spectrum prophylactic vaccine against both T3SA^+^ (type III secretion apparatus) and ExlA^+^/T3SA^-^ Pa strains worsen the situation in a post-pandemic world. Thus, we formulated a candidate subunit vaccine (called ExlA/L-PaF/BECC/ME) against both Pa types. This bivalent vaccine was generated by combining the C-terminal active moiety of exolysin A (ExlA) produced by non-T3SA Pa strains with our T3SA-based vaccine platform, L-PaF, in an oil-in-water emulsion. The ExlA/L-PaF in ME (MedImmune emulsion) was then mixed with BECC438b, an engineered lipid A analogue and a TLR4 agonist. This formulation was administered intranasally (IN) to young and elderly mice to determine its potency across a diverse age-range. The elderly mice were used to mimic the infection seen in elderly humans, who are more susceptible to serious Pa disease compared to their young adult counterparts. After Pa infection, mice immunized with ExlA/L-PaF/BECC/ME displayed a T cell-mediated adaptive response while PBS-vaccinated mice experienced a rapid onset inflammatory response. Important genes and pathways were observed, which give rise to an anti-Pa immune response. Thus, this vaccine has the potential to protect aged individuals in our population from serious Pa infection.

## Introduction

The opportunistic pathogen *Pseudomonas aeruginosa* (Pa) causes severe hospital-acquired infections in immunocompromised individuals. Treating Pa infections has become increasingly difficult due to innate and acquired antibiotic resistance. Multidrug resistant (MDR) Pa strains are now classified as serious threats by the CDC (1) (https://www.cdc.gov/drugresistance/biggest-threats.html#pse). Both MDR and XDR (extremely drug resistant) Pa impose a massive healthcare burden globally (2). Pa causes severe ventilator-associated pneumonia (VAP) in immune-compromised individuals under life support settings as well as patients with burns, severe wounds, or catheterization (urinary tract infections) (3, 4). While in healthy young adults, Pa typically results in asymptomatic, self-limiting infections, it can cause lethal infections in an aging population. Moreover, all individuals with cystic fibrosis are at an increased risk irrespective of their age (5). Limited therapeutic interventions and the high cost of medical treatment and hospitalization make prophylactic approaches a top priority.

Vaccination is among the best ways to prevent infectious diseases. Historically, live-attenuated and whole cell immunogens have been used successfully, however, they can cause unwanted side effects, especially in immunocompromised individuals (6, 7). LPS-containing killed whole cell and outer membrane protein vaccines can also be efficacious to some extent, but can be reactogenic and often they do not provide the adequate broad protection that is needed. In the absence of a licensed vaccine against this pathogen, our group has focused on developing effective subunit protein vaccines that target the type III secretion apparatus (T3SA) possessed by important enteric and respiratory pathogens (7–12).

Previously, we described an oil-in-water emulsion-based vaccine containing a fusion protein generated from LTA1 (a mucosal adjuvant derived from the double-mutant labile toxin (dmLT) from enterotoxigenic *E. coli*) with the Pa T3SA proteins PopB and PcrV (L-PaF). L-PaF was formulated in the context of the squalene-based MedImmune Emulsion (ME) and the lipid A analogue BECC438b (to give L-PaF/BECC/ME). This formulation was found to be efficacious in young inbred and outbred mice (8–10) in which it elicited a strong adaptive response against clinical Pa strains belonging to serogroups 04 and 06. The major shortcoming of L-PaF/BECC/ME was its inability to generate a protective response against T3SA^-^/exolysin A^+^ (ExlA^+^) PA7-like clade (serogroup O12) of Pa. Thus, an unmet need is to target the ExlA^+^ strains, thereby generating a broad-spectrum immunogen against Pa (13–16).

In the present study, ExlA has been formulated with the L-PaF/BECC/ME platform. The protective efficacy of this immunogen was assessed in young and elderly mice where it elicits humoral and cellular immune responses that protect mice against Pa infections by T3SA^+^/ExlA^-^ and T3SA^-^/ExlA^+^ strains. Follow up mRNA studies of the elderly mouse lung reveal the association of Th17, Th1/Th2, and TCR (T cell receptor) signaling pathways with protection, along with an elevated mucosal immune response. Moreover, the presence of a strong innate response post-challenge is associated with an unfavorable outcome. Thus, the L-PaF/ExlA immunogen has the potential to be used as a broad-spectrum anti-Pa immunogen in future studies.

## Materials and methods

### Materials

Squalene was purchased from Echelon Biosciences (Salt Lake City, UT). dmLT was a gift from Elizabeth Norton, Tulane University School of Medicine (New Orleans, LA). All other reagents and chemicals were from Millipore-Sigma Chemical Co. (St. Louis, MO) or Thermo-Fisher Scientific (Waltham, MA).

## Methods

### Protein and formulation

L-PaF, BECC438b, and ME were prepared as previously described (9, 10). The sequence for the C-terminus of ExlA (residues 1361-1651) was cloned into pET-15b to be expressed with an N-terminal histidine tag (HT). After sequencing to confirm the DNA sequence, the plasmid was transformed into *E. coli* BL21(DE3) for over-expression of ExlA. An overnight culture was grown in LB containing ampicillin at 37°C. A 2 mL aliquot of this culture was added to each of eight Fernbach flasks containing 1L of LB with 100 µL ampicillin to an A_600_ of 0.05. The flasks were then incubated at 37°C with shaking at 200 rpm to an the A_600_ of 0.8. IPTG was added to 1 mM and the bacteria incubated another 3 h. The bacteria were collected by centrifugation, resuspended in IMAC binding buffer (20 mM Tris pH 8.0, 500 mM NaCl, 10 mM imidazole), lysed by sonication and the solution clarified by centrifugation at 22,000 x g for 30 min at 4°C. The supernatant containing the HT-ExlA was loaded onto a 5 ml IMAC cartridge, and the cartridge washed with 30 column volumes of binding buffer. After elution with IMAC elution buffer (20 mM Tris pH 8.0, 500 mM NaCl, 500 mM imidazole), HT-ExlA was dialyzed against PBS with one buffer change. LPS was removed from all proteins using Triton X-114 phase separation. Triton X-114 was added to 1%, vortexed, incubated on ice for 5 min. The solution was then incubated at 37°C for an additional 5 min. Finally, the phase-separated solution was clarified for one minute at 13,000 x g. The upper phase containing HT-ExlA was decanted, and endotoxin levels measured using a NexGen PTS with EndoSafe cartridges following manufacturers protocols (Charles River Laboratories, Wilmington, MA). The Triton phase separation was repeated, if needed, until the endotoxin activity measured < 5 endotoxin unit/mg protein.

### Mouse immunization and sample collection

BALB/c mice were acquired from Charles River Laboratories (Wilmington, MA). Two age groups of mice were vaccinated. One age group consisted of young adults (6 – 8 weeks old), while the other group were aged in-housed to 24 months of age and were considered elderly. The animals (n = 10) in these groups were immunized as follows: (a) PBS; (b) ExlA + dmLT; (c) ExlA + L-PaF + BECC/ME; (d) L-PaF + BECC/ME; (e) ExlA alone; (f) L-PaF; (g) PaF ± dmLT. For ExlA, dose escalation was conducted, while the doses for L-PaF, ME, and BECC438b were previously optimized (8–10). Mice were bled on days 0 and 56 post first immunization. Additionally, on day 56, both lungs from five mice were harvested to assess pre-challenge immune status. Finally, mice (n=5) were challenged with either CEC124 (ExlA^+^) or mPa08-31 (T3SA^+^), both of which are Pa clinical isolates. Post-challenge mouse lungs were harvested on either 1 day (if challenged with CEC124), or 2 days post infection (DPI) (if challenged with mPa08-31) to enumerate bacterial burden and immune correlates of protection.

Elderly mice were immunized with either PBS or ExlA + L-PaF + BECC/ME and challenged as described above. Their lungs were subjected to bulk mRNA analysis before and after challenge.

### Ethical statement

All animal studies were carried out in accordance with the IACUC animal use statement (AUS 222-03) from the University of Kansas.

### IgG and IgA responses

To assess the immune responses against each immunogen component (PcrV, PopB, and ExlA), 96-well microtiter plates were coated with 1 µg/ml of PcrV, PopB, or ExlA. Following coating, the plates were blocked with 10% non-fat dry milk overnight at 4°C. Subsequently, mouse sera collected on day 56 post-first immunization were added. HRP-tagged secondary antibodies were then employed: anti-mouse IgG (1:1000, cat. # 5450-0011 (474-1806) Sera Care, USA) or anti-mouse IgA (1:4000, cat. # OB1040-05, Southern Biotech, USA). TMB was used as the substrate and the reaction was stopped with phosphoric acid. Endpoint titers were measured as EU/ml.

### Bacterial culture and mouse challenge

The Pa strains CEC124, and mPa08-31 were cultured overnight at 37°C with shaking at 200 rpm. They were then inoculated into fresh low-salt LB medium at a 1:100 ratio. Bacterial cultures were cultured until reaching an optical density at 600 nm (A600) of 0.3. The CEC124 strain was then adjusted to a concentration of 1 x 10^7^ CFU/30 µl, and mPa08-31 to a concentration of 4 x 10^7^ CFU/30 µl. Mice were anesthetized using isoflurane and then challenged intranasally (IN) at least 56 days after their first immunization. Each mouse received 30 µl of one of the bacterial suspensions (n=5). CEC124 challenged mice lungs were harvested on 1 day post infection or DPI (approximately 16 – 18 hours post infection or HPI), and mPa08-31 challenged mice lungs on 2 DPI. For mRNA seq analyses, separate sets of elderly mice were used (n=3). Data were represented as from n=2 due to the large amount of data obtained using these assays. All conditions were identical throughout the experiment.

### Organ processing

Mouse lungs were collected into conical tubes containing MACS tissue storage solution and later processed with a lung dissociation kit (Miltenyi Biotec, Gaithersburg, MD). The red blood cells present were lysed, and the cell suspension was adjusted to a concentration of 1 x 10^7^ cells/ml. Single cell suspensions were used for ELISpot, MSD cytokine analyses (MesoScale Discovery, Rockville, MD), and bacterial enumeration.

### ELISpot assay

Lung cells were incubated at 37°C for 24 h with or without additional stimulation with one of the three proteins (PcrV, PopB, ExlA) at a concentration of 5 µg/ml. Dual color ELISpot plates were coated with capture antibodies against IFN-γ and IL-17 for the downstream enzymatic assay according to the manufacturer’s instructions (ImmunoSpot, Shaker Heights, OH). Spot forming units were quantified using the CTL analyzer as per the manufacturer’s specifications.

### Total cytokine secretion from isolated cells

Lung cells were incubated at 37°C for 48 h in the presence or absence of the proteins (PcrV, PopB, ExlA) at a concentration of 10 µg/ml. The supernatant fractions were then collected and analyzed using U-PLEX kits for the quantification of IL-2, IFN-γ, IL-6, IL-17A, and TNF-α. The cytokine concentrations were measured using an MSD plate reader as per manufacturer’s specifications.

### mRNA sequencing

Single cell suspensions of lung cells were used for all sequencing studies. Mice were challenged with either CEC124 or mPa08-31 prior to the collection of the lungs. RNA was isolated using the RNeasy plus Universal mini kit (Qiagen, Germantown, MD). An RNA integration number of seven or higher was considered for downstream processing. mRNA sequencing was conducted at Novogene Ltd.

### Treatment of the mRNA sequencing data

The total read counts were subjected to data processing and differential expression analyses using iDEP (17). Cutoff values for positive counts were set as described before (10). Briefly, at least 0.5 counts/million (CPM) reads were considered and transformed into log_2_(CPM) by EdgeR. DESeq2 was used to identify the differentially expressed genes with a minimum fold-change of 2. The enriched pathways were identified through Gene Ontology (GO) analysis. Heatmaps, dotplots, and cnet plots were prepared based on the manually selected immunological pathways.

### Data and statistical analysis

Data processing and statistical analyses were performed using GraphPad Prism v8.1.2, R programming and Metaboanalyst v5.0. Data followed a non-parametric distribution. Normality was checked by Sapiro-Wilk test. For comparisons involving three or more groups, Kruskal-Willis test with Dunn’s multiple comparison *post hoc* test was employed. If more than one variable was shown in a single graph, one variable was analyzed (PcrV-treated, PopB-treated etc.) at a time. When analyzing two groups, Mann-Whitney U test was applied. PBS was used as control in all cases.

To determine unique genes with 10-fold or higher (upregulation) and 5-fold or lower (downregulation), genes were further filtered based on exclusion of the term ‘GM.’ Except for elderly mice pre-challenge (n=4), all other studies were conducted with n=5 mice/group. In figure legends, *p*-values were reported, with significance levels indicated as follows: *p < 0.05, **p < 0.01, ***p < 0.001, ****p < 0.0001.

## Results

### ExlA alone or in combination with L-PaF ± BECC/ME is immunogenic

**Figure 1:**
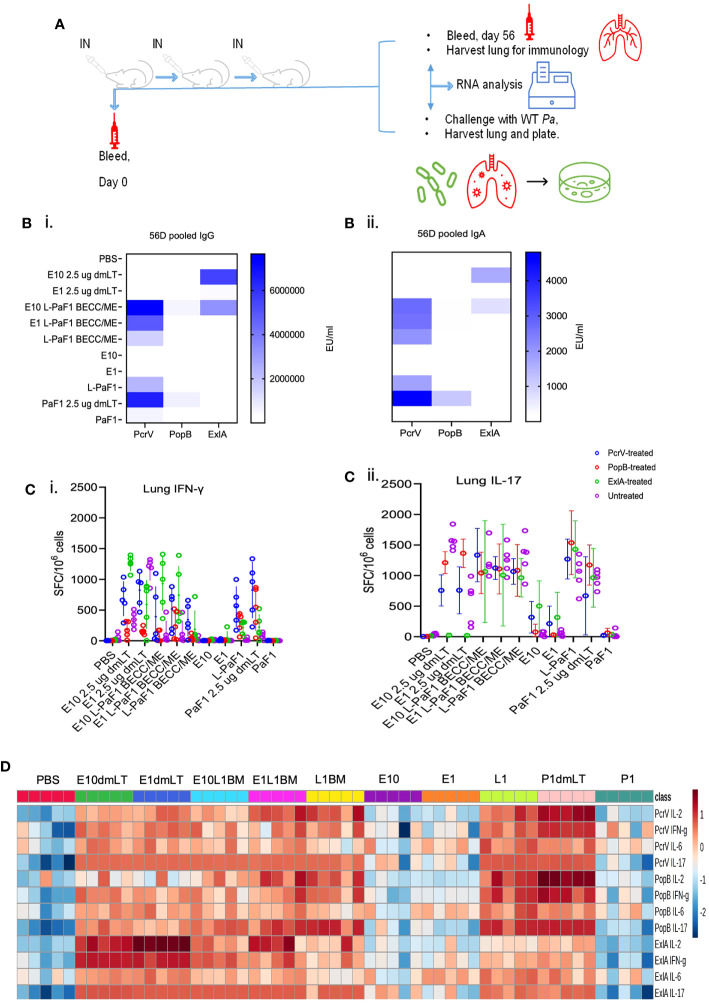
Experimental design, and immunogenicity studies in young adult mice. **(A)** BALB/c mice were vaccinated on days 0, 14 and 28 and sera collected on days 0 and 56 post first immunization. Pre-challenge immunology was carried out by ELISpot, MSD, and mRNA seq on samples from day 56. For post-challenge studies, mice were challenged on day 56 with lung cells collected for bacterial enumeration, immunological assays, and mRNA seq. **(B)** Serum IgG **(Bi)**, and IgA **(Bii)** levels were assessed with pooled serum against each of the proteins. **(C)** (ELISpot), and **(D)** (MSD). Cell suspensions were prepared and stimulated with one of the three proteins or left untreated. At 24 h, IFN-γ **(Ci)**, and IL-17A **(Cii)** secreting lung cells were enumerated, whereas, at 48 h, the total amounts of secreted IL-2, IFN-γ, IL-6, and IL-17A were quantified by MSD. Values in **(Ci, ii)** were plotted as individual points ± SD (n = 5/group). Error bars represent SD. Heatmap in **(D)** was generated with each biological replicate (n = 5/group). Groups have been denoted on the top of the heatmap. E10dmLT = E10 2.5 μg dmLT, E1dmLT = E10 2.5 μg dmLT, E10L1BM = E10 L-PaF1 BECC/ME, E1L1BM = E1 L-PaF1 BECC/ME, L1BM = L-PaF1 BECC/ME, E10 = same as before, E1 = same as before, L1 = L-PaF1, P1dmLT = PaF1 2.5 μg dmLT, and P1 = PaF1. Statistical significance was calculated as mentioned in the text. The detailed statistical analyses can be found in **Supplementary Tables 1 and 2**. Graphical representation of the heatmap can be found in **Supplementary Figure 1** (top panel).

Mice were vaccinated IN with PBS or different formulations containing L-PaF and/or ExlA using the schedule shown in **Figure 1A**. Serum IgG and IgA levels against each protein (PcrV, PopB and ExlA) were assessed in serum collected on day 56 post first immunization. Anti-PcrV and anti-PopB IgG and IgA were observed in mice immunized with L-PaF ± ExlA/BECC/ME, whereas anti-ExlA IgG and IgA were observed in all groups vaccinated with ExlA (**Figures 1B**i, ii). The anti-PopB response was found to be lower than that for anti-PcrV, which is typical of the results seen in our previous studies involving PaF and L-PaF (9, 10).

Cytokine secreting cells and total cytokine secretion from isolated lung cells were measured using ELISpot and MSD assays, respectively. Lung cells were isolated and stimulated individually with each of the proteins (PcrV, PopB, and ExlA) or left unstimulated. At 24 h, the number of lung cells secreting IFN-γ and/or IL-17 were enumerated via ELISpot (**Figures 1C**i, ii). The unstimulated groups showed very few cytokine secreting cells compared to the PcrV-, PopB- and ExlA-stimulated groups. Mice vaccinated with ExlA with dmLT had an IFN-γ response that was higher than the other vaccinated groups (**Figure 1C**i), but it did not reach statistical significance (**Supplementary Tables 1A–D**). In contrast, the numbers IL-17 secreting cells were comparable across the vaccinated groups (**Supplementary Tables 1E–H**). The groups vaccinated with E10 (10 µg ExlA) + dmLT, E1 (1 µg ExlA) + L-PaF1 (1 µg L-PaF) + BECC/ME or L-PaF1 + BECC/ME showed significantly greater IL-17 responses after ExlA stimulation compared to the PBS vaccinated group. PcrV-stimulated lung cells showed significantly higher IFN-γ responses in the presence of dmLT + ExlA. E1 + L-PaF1 + BECC/ME and PaF ± dmLT vaccinated groups when compared to the PBS vaccinated group (**Figure 1C**i). PopB stimulation resulted in significantly higher IFN-γ responses in the E1 + L-PaF1 + BECC/ME and PaF ± dmLT vaccinated groups (**Figure 1C**i). ExlA stimulation resulted in significantly higher IFN-γ in all the ExlA vaccinated groups (**Figure 1C**i). Comparable numbers of IL-17 secreting lung cells were seen in groups vaccinated with ExlA/L-PaF combinations regardless of the protein used for stimulation (**Figure 1C**ii).

**Figure 2:**
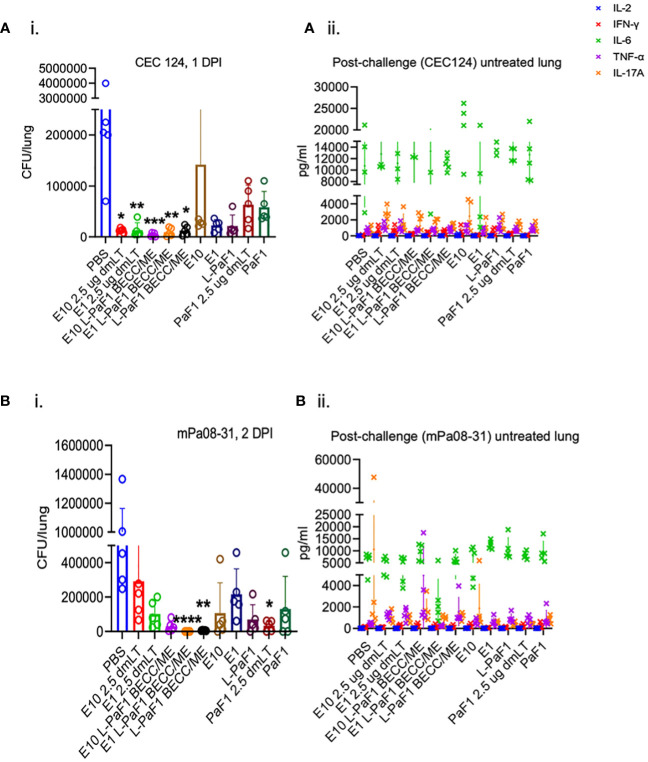
Protective efficacy studies in young adult mice. BALB/c mice were challenged with either **(Ai)**. CEC124 or **(Bi)**. mPa08-31 with doses stated in the text. Mouse lungs were harvested post-challenge and total cytokine secretion was quantified for the CEC124 **(Aii)** and mPa08-31 **(Bii)** challenge. Values were plotted as individual points ± SD (n = 5/group). Error bars represent SD. Statistical significance was calculated as stated in the text. Detailed statistical analyses on cytokine secretion can be found in **Supplementary Table 3**.

MSD analysis was used to quantify the total cytokines secreted by isolated cells. Lung cells were incubated with or without protein stimulation and then incubated for 48 h and the supernatant fraction collected for analysis. Stimulation with PcrV resulted in the enhanced secretion of most of the cytokines, PopB or ExlA stimulation resulted in modest IFN-γ, IL-6 and IL-17A secretion (**Figure 1D** and **Supplementary Figure 1** top panel). The statistical significance tables can be found in the **Supplementary Tables 2A–L**).

### The ME (nanoemulsion) vaccine protects young adult mice against challenge with Pa clinical isolates

The protective efficacy of the formulations was assessed via bacterial challenge with clinical isolates of Pa - CEC124 (a T3SA^-^/ExlA^+^ Pa strain) or mPa08-31 (a T3SA^+^/ExlA^-^ Pa strain). Both lungs were collected from each mouse at 1 DPI and 2 DPI, respectively, for CFU enumeration and determination of cytokine secretion. Mice vaccinated with ExlA + dmLT or ExlA + L-PaF BECC/ME and challenged with CEC124 exhibited the lowest bacterial burden compared to the PBS vaccinated group (**Figure 2A**i). Elevated IL-6 and IL-17A was observed in all mice after challenge with Pa CEC124 regardless of the groups (**Figure 2A**ii). When challenged with mPa08-31, the E1 + L-PaF1 + BECC/ME, L-PaF1 + BECC/ME and PaF1 + dmLT vaccinated mice showed a significant reduction in bacterial burden compared to the other groups (**Figure 2B**i), which was accompanied by relatively higher IL-6 secretion from the isolated lung cells (**Figure 2B**ii). Details of the statistical analyses are presented in **Supplementary Tables 3A–J**. Correlation between lung burden and post-challenge cytokines was then calculated. While not statistically significant, post-challenge IFNγ and TNFα had a strong negative correlation trend with lung burden, while IL-6 and IL-17A had a positive correlation trend with lung burden (**Supplementary Figure 2**). Statistically significant positive correlation was found between pre-challenge lung IL-6, IL-2 and CFU burden (**Supplementary Figure 1**, bottom panel and **Supplementary Table 4**).

### The E1 + L-PaF1 + BECC/ME nanoparticle vaccine best protects elderly mice from clinical Pa isolates

**Figure 3:**
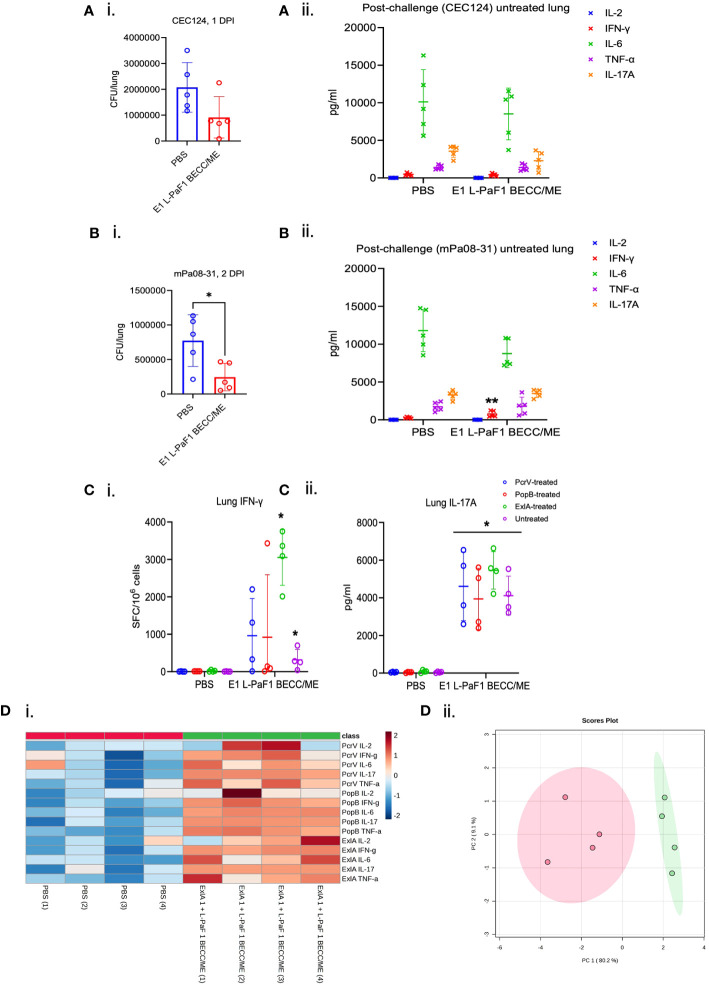
Immunogenicity and protective efficacy in elderly mice. Elderly (> 24-month-old) mice were immunized following the same scheme, but with only two vaccine formulations, PBS or E1 + L-PaF1 + BECC/ME. These mice were challenged with either CEC124 **(Ai)** or mPa08-31 **(Bi)**. The lungs were then harvested, bacterial burden determined by dilution plating. Post-challenge cytokines from CEC124 **(Aii)** challenged and mPa08-31 **(Bii)** challenged mouse lungs were measured by MSD assay. Pre-challenge lung cytokine secreting cells were measured by ELISpot (**Ci** for IFN-γ and **Cii** for IL-17A) with or without protein stimulation. Pre-challenge lung total cytokines were measured by MSD assay **(Di, ii)** with protein stimulation. Values in **(A–C)** were plotted as individual points ± SD. n = 5/group in post-challenge studies, i.e. **(A, B)** n = 4/group in pre-challenge studies, i.e., **(C, D)** Error bars represent SD. Heatmap in **(Di)** was generated with each biological replicate (n = 4/group). The name of the group has been denoted at the bottom. Name of the protein the cells were stimulated with, along with the name of the cytokines were denoted at the right side of the heatmap. Scores plot in **(Dii)** was created by Metaboanalyst 5.0 to show differences among the groups. Red dots denote PBS group and green dots denote ExlA1 L-PaF1 BECC/ME group. Statistical significance was calculated as mentioned in the text. *p < 0.05.

People are exposed to Pa throughout their lives, but an actual infection is not established with the immune system rapidly clearing the organism. For the elderly population, however, Pa may cause an acute and/or chronic infection. To ascertain the potential for the ExlA/L-PaF platform to be effective in an elderly population, we vaccinated elderly mice with E1 + L-PaF1 + BECC/ME based on observations in the young adult mice and assessed the protective efficacy. Twenty-four-month-old mice were used which corresponds to 69 years in human age term. These mice were challenged with CEC124 or mPa08-31. Lung burden in immunized mice was much lower than in the PBS immunized groups for both Pa strains (**Figures 3A**i, **B**i). Post-challenge IL-6 and IL-17A levels were elevated in both challenge groups (**Figures 3A**ii, **B**ii) with IFNγ being significantly higher in the immunized group for the mPa08-31 challenged mice. In contrast, levels of post-challenge IL-2, IL-17A and TNF-α levels were comparable between the challenge groups. Mouse lung cells were also assessed for the numbers of cytokine secreting cells, as well as the total amount of cytokines being secreted before challenge. ELISpot assays showed a response similar to that seen for the younger mice, where the immunized group was found to be secreting statistically high levels of IFN-γ and IL-17 (**Figures 3C**i, ii). MSD assays showed pre-challenge lung cells to be secreting considerably higher amounts of IL-2, IFNγ, IL-6, IL-17A, and TNFα in the immunized group compared to their PBS vaccinated counterparts (**Figure 3D**i). Principal component analysis showed two different populations for the control and immunized groups (**Figure 3D**ii).

### CEC124 and mPa08-31 follow similar pathways to establish infection in the lungs

The pathogenesis of a bacterium is often dependent upon how the host responds to the presence of the pathogen. To determine host responses to Pa infection in young adult and elderly mice, mRNA sequencing of the lung cells from mice challenged with either the T3SA^-^ CEC124 or T3SA^+^ mPa08-31 strain was carried out and showed that several common pathways were observed and were present after both challenges (**Supplementary Figures 3A–D**). A total of 2078 and 2345 genes were shown to be upregulated and 593 and 455 genes were downregulated by at least two orders of magnitude in the CEC124 and mPa08-31 challenged mice, respectively, when compared to their uninfected controls. Pathways related to cytokine-cytokine receptor interactions, neutrophil extracellular trap formation, chemokine signaling, TNF, TLR, Th17, IL-17 signaling, necroptosis, and C-type lectin receptor signaling were upregulated in mice infected with either CEC124 or mPa08-31 when compared to uninfected controls. No common pathways were observed that were downregulated regardless of the Pa strain (**Supplementary Figures 3A–D**).

At the genetic level, a cut-off value of 10-fold or more for upregulation and 5-fold or more for downregulation was used. Based on the obtained values, several unique genes were found to be important for Pa pathogenesis (**Supplementary Figure 4**, top panel and **Supplementary Table 5**). The genes that stood out as affected most with large up- and down-regulation considered were from chemokine families, carbohydrate metabolism, G protein coupled receptors, metal binding, ion channel regulators, and a few others. When compared, the gene up/down-regulation profile was altered for the unimmunized/infected versus immunized/infected mice (**Supplementary Figure 5**). These genes belong to various pathways with 32 of those pathways overlapping for both strains (**Supplementary Figure 4**, bottom panel). Immunized/infected groups showed a more controlled cytokine response, whereas a hypoxia-mediated cytokine storm was observed in the unimmunized/infected mice. An extended version of this heat map is presented as **Supplementary Figure 6**.

### Immunization with E1 + L-PaF1 + BECC/ME invokes T cell mediated adaptive immunity

**Figure 4:**
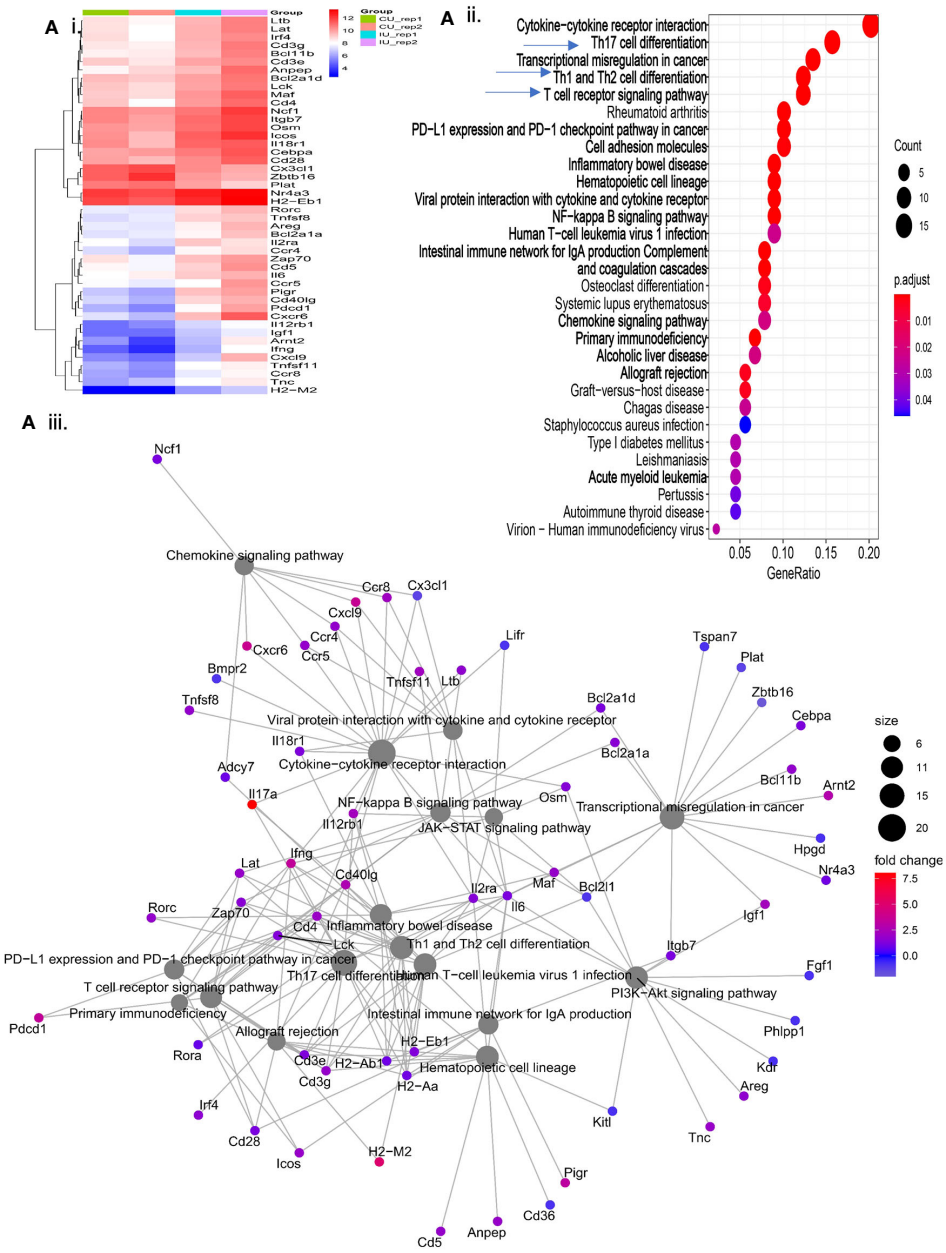
Evaluation of immunologically relevant genes and pathways in immunized mice. Elderly mice were immunized with PBS or E1 + L-PaF1 + BECC/ME on days 0, 14 and 28. On day 56, the lungs were collected, single cell suspensions were prepared and RNA was isolated. In **(Ai)**, the heatmap depicts the nature of up/downregulation of genes in immunized mice lung compared to the PBS (control) mice. An extended version of the same map can is shown in **Supplementary Figure 5**. In **(Aii)**, Dotplot depicts the pathways up/downregulated in immunized mice lung compared to the PBS (control) mice. In **(Aiii)**, cnet plot depicts the different interconnected pathways and how are they either up- or down-regulated in the immunized mouse lung compared to the PBS (control) mouse lungs. CU = Control (PBS), IU = Immunized. Rep = Biological repeats.

Our previous work has focused on young adult mice, however, the Pa incidence rate in the elderly population represents a more immediate public health concern. With that in mind, we assessed the efficacy of our vaccine in both young adult and elderly mice followed by an in-depth analysis in elderly mice. Three doses of E1 + L-PaF1 + BECC/ME elicited up-regulation of an array of genes related to different immune pathways (**Figure 4A**i). Dot plot analysis of these genes illustrate up- or down-regulated pathways in immunized mice compared to unimmunized mice (**Figure 4A**ii). The cnet plot depicts how these pathways are connected to each other through common genes (**Figure 4A**iii). Differentiation of T cells to Th17 is supported by the up-regulation of *il17a*, along with Rorc, Th1, Th2 and TCR. Additionally, cytokine-cytokine receptor interaction pathways had a greater number of activated genes compared to the other pathways. T cell mediated adaptive immunity was mounted by the co-stimulatory marker CD28 in immunized mice, along with downstream T cell response genes, Lat, Lck, and Zap70. The mucosal immune response was found to be activated to produce secretory IgA. This anti-Pa response was initially facilitated by NF-κB and multiple chemokine signaling pathways. The presence of chemokines such as Ccr4, Ccr5, Cxcl9 is an indication of their importance in the generation of the immunity.

### T cell mediated immunity protects the elderly mice from Pa

**Figure 5:**
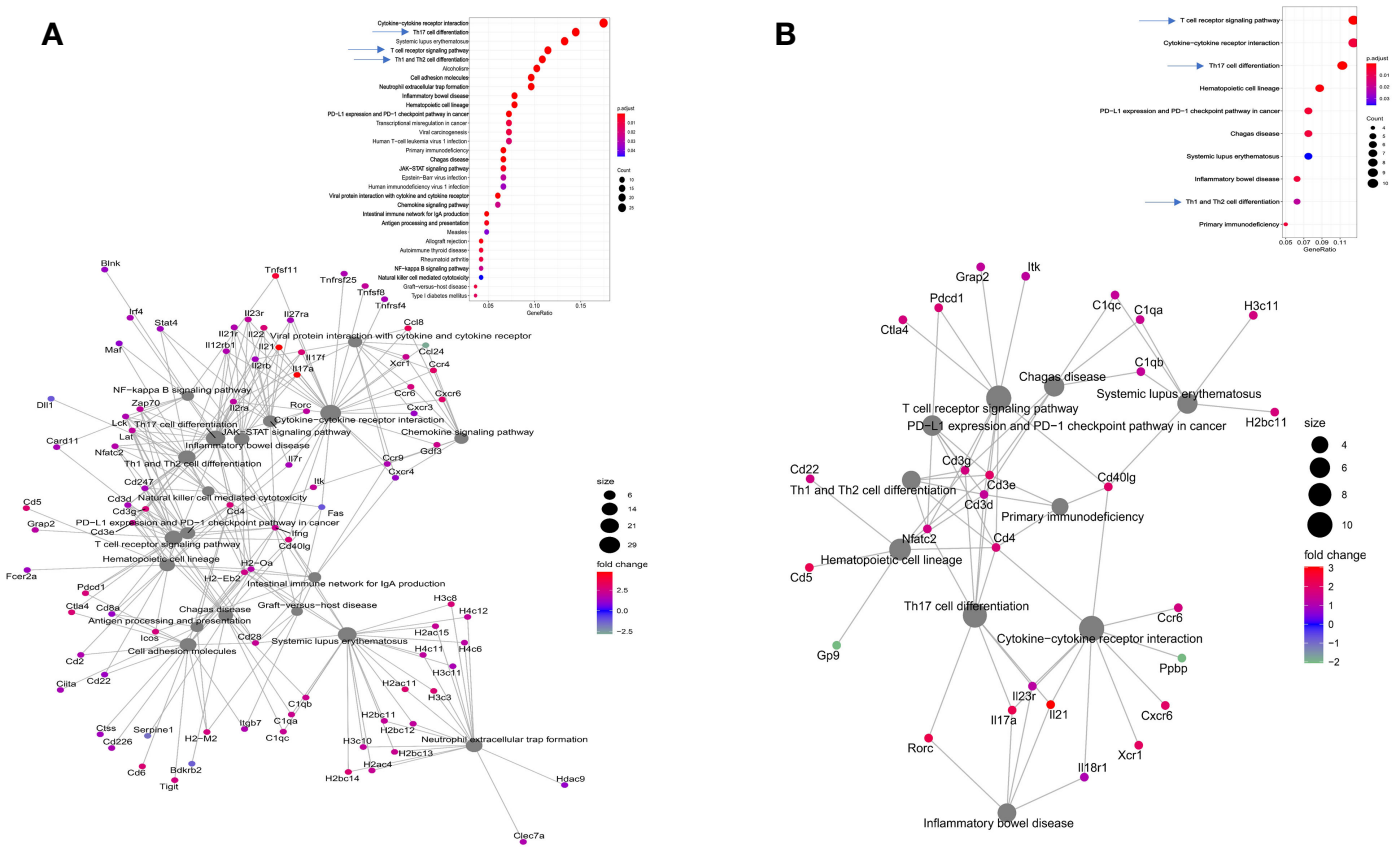
Consequences of Pa challenge in mice lung. Elderly mice were immunized and challenged with either CEC124 **(A)** or mPa08-31 **(B)** as stated in the text. The lungs were harvested for RNAseq sequencing and analysis. The top panel shows differentially up- or down-regulated pathways in the immunized/infected mice compared to the PBS vaccinated/infected mice. The bottom panel shows the connections between those pathways and their corresponding genes, either up- or down-regulated, through a cnet plot.

**Figure 6:**
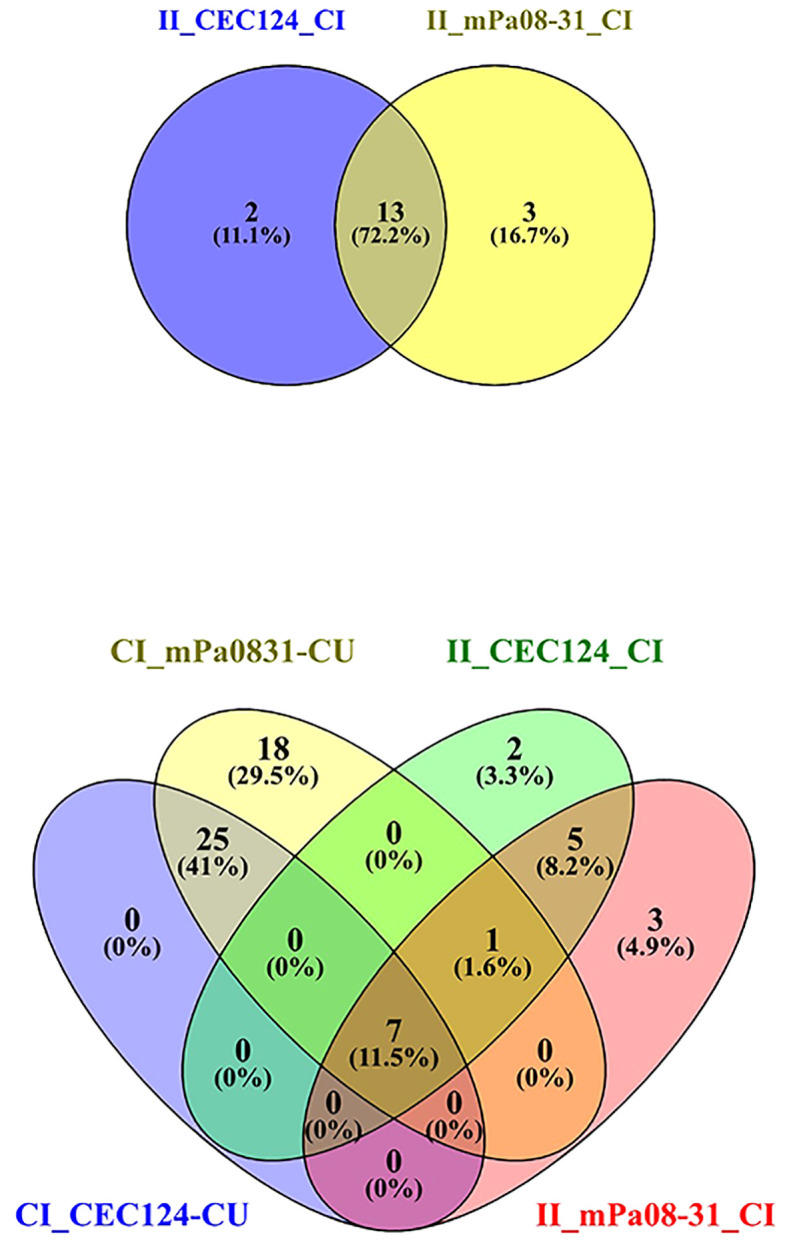
Venn diagram depicts common pathways among different conditions. Elderly mice were challenged, and lungs collected as previously stated. The top panel shows the number of common pathways in immunized/infected CEC124 and immunized/infected mPa08-31 mice lungs. The bottom panel shows the number of common pathways in control/infected, immunized/infected and control/uninfected mice lungs. CI_CEC124_CU = Control infected with CEC124 over control uninfected; CI_mPa08-31_CU = Control infected with mPa08-31 over control uninfected; II_CEC124_CI = Immunized infected with CEC124 over control infected; II_mPa08-31_CI = Immunized infected with mPa08-31 over control infected. The numbers represent the actual number of pathways in each condition.

The elderly mice were challenged with either CEC124 or mPa08-31. The lungs were collected on 1 DPI, and 2 DPI, respectively. Immunized/infected mice lung exhibited a distinct response to the infection compared to the PBS vaccinated mice. As seen in immunized/uninfected mice, the immunized/infected mice had experienced a T cell mediated response corresponding to Th17, Th1, Th2, TCR signaling pathways (**Figures 5A, B**, top panels). When challenged with CEC124, the elevated levels of *il17a*, *il17f*, *il17r*, *il21*, *il21r*, *il22*, *il23r*, and *Rorc* observed in the immunized mice indicates the involvement of a Th17 response in mounting an anti-Pa immune response (**Figure 5A**, bottom panel). Additionally, infγ, *cd4*, *ctla4*, *cdba*, and *Nfatc2* were observed as being up-regulated among others. The presence of these genes shows the involvement of IFN-g with activated CD4 T cells. The presence of cdba or activation induced cytidine deaminase hints further class switching in the germinal centers post infection. The presence of costimulatory molecules, cell adhesion molecules, and involvement of innate immune response gene expression (e.g. neutrophil extracellular trap, NF-κB and chemokines) were observed. The intestinal immune network for IgA production was also found to be elevated (**Figure 5A**, bottom panel). Since the mucosal system is intertwined, elevation of IgA in the intestinal tract may help generate IgA in the bronchoalveolar space as well, which was not assessed. The presence of such an extensive number of genes was not seen in mPa08-31 infected mice (**Figure 5B**, bottom panel). When CEC124 and mPa08-31 infected immunized elderly mice were compared, thirteen pathways seemed to be important to confer an anti-Pa immune response (**Figure 6**, top panel). Most of the genes belonged to Th17, Th1/Th2, TCR, cytokine-cytokine receptor signaling pathways. When unimmunized/infected and immunized/infected were compared, five pathways appeared essential to control the Pa infection (**Figure 6**, bottom panel), which include TCR, Th1/Th2 and cell adhesion molecules. Th17 was also found to be important, as was seen for the unimmunized post-challenge mPa08-31 mice. However, unimmunized CEC124 post-challenge mice did not give rise to any Th17 response. Twenty-five pathways seem to have contributed to pathogenicity post-challenge including IL-17, TNF, NOD-like molecule signaling pathway, chemokine, necroptosis, TLR signaling, as well as others. While the immunized mice cleared the infection with the help of T cell-mediated immunity, the unimmunized mice succumbed to it.

## Discussion

Pa is an opportunistic pathogen that imposes a great risk to the immunocompromised, including our aging population. Cystic fibrosis patients are also at grave risk from acute Pa infection that can result in chronic, host-adapted colonization, but this organism can also cause meningitis, urinary tract infections, community-acquired pneumonia, ocular infections, and even death in some cases. One study indicates the risk of illness per event based on disability adjusted life year (DALY) to be 2.81 x 10^-9^ (18). Increasing innate and acquired antibiotic resistance and the lack of a licensed vaccine makes the scenario challenging to the healthcare workers. Among the 20 known serogroups, O11 is the most prevalent serogroup in population studies, followed by O6, O1 and O12. O12 is of particular importance because of its abundance in hospital-acquired cases and increasing drug resistance (14). Young adult and older populations contract Pa throughout their lives, however, the elderly are more prone to develop overt disease. While Pa is typically asymptomatic or self-limiting in the young, immune senescence in the elderly results in a reduction in both B and T cells in the bone marrow and thymus, respectively, as well as a reduction in the function of the mature lymphocytes in the secondary lymphoid tissue. This reduced immune responsiveness increases Pa’s ability to colonize the upper and lower respiratory track, resulting in an increased risk of fulminant disease in the elderly (19). CDC data implicate the elderly population (≥ 65 years) to be hospitalized more often than the younger adults (18 – 64 years) due to Pa infection. Since Pa is a nosocomial pathogen, longer stays in the hospital for the elderly increases the chances of being infected (18).

Recently, our group developed a T3SA subunit vaccine, L-PaF/BECC/ME, that we have demonstrated to be protective against serogroups O4 and O6, however, this vaccine would be unable to stop the infection from O12 strains of the PA7 clade since they do not have a T3SA. These PA7-like strains (and the PA39 clade) possess the *exlA* gene, which is part of the ExlAB two partner secretion system. Moreover, 30 – 50% of clinical isolates are phenotypically T3SA^-^ (15). With this in mind, we have developed a multivalent protein subunit vaccine consisting of L-PaF and ExlA, which make it effective against a broad range of Pa. We formulated it as an oil-in-water, squalene-based nanoemulsion (ME) and added the lipid A analogue and TLR4 agonist BECC438b to further boost vaccine efficacy (20). The efficacy of this new vaccine, ExlA + L-PaF + BECC/ME, was assessed in young adult and elderly mice here to shown that it is immunogenic, giving rise to a T cell mediated adaptive response that controls the onset of inflammatory responses caused by infection in both populations. In the absence of the adaptive immune response elicited by the vaccine, the control (PBS vaccinated) mice became morbid.

Previously, we demonstrated the importance of having a systemic humoral response, and a local lung response, to mount an effective anti-Pa immune response (10). The presence of serum IgG and IgA against PcrV and PopB of the Pa T3SA confirms the presence of a systemic immune response in the immunized mice. Moreover, upregulation of the intestinal immune network for IgA production (**Figure 4A**ii), which is an important component of the common mucosal immune system (CMIS), indicates the involvement of the local response as well. CMIS is regulated by the association and location of the MALT (mucosa associated lymphoid tissue). They contain lymphoid cells that are diffusely located throughout the parenchyma of the mucosal organs to act as effector sites where the immune responses are manifested (21). In addition to the humoral response, a strong cellular response was seen in the mice immunized here, which was either absent or downregulated in the PBS vaccinated mice. The immunogen used here seems to sensitize the antigen-presenting cells (APCs) at the mucosal inductive sites, which then moves into the lymph, enters the circulation, and ultimately seeds the effector sites with the help of their homing receptors.

The presence of a strong T cell response in the form of Th1/Th2, and Th17 appears to be important in the generation of the anti-Pa immunity. The PAMP (pathogen associated molecular pattern) hypothesis of differential generation of Th1 and Th2 suggests that activation of CD4 T cells require the presence of PAMPs to activate the APCs to express costimulatory molecules (CoS). The cytokine milieu hypothesis, on the other hand, suggests the presence of certain cytokines with a type-specific response is important for protection. In this case, the presence of IFN-γ dictates a Th1 response while IL-17A corresponds to Th17. Although both Th1 and Th2 responses are consistently observed in our studies, the Th1 response is typically stronger than the Th2 response. This distinction might be explained by the threshold hypothesis which dictates that the amount of antigen and the antigen interaction time between CD4-activated B cells and naïve CD4 T cells determines the outcome of an immune response. A minimal amount of antigen corresponds to a “lower strength of cooperation” resulting in a Th1-biased immune activation, while a greater concentration of immunogen causes the opposite resulting in a Th2 response. Dose escalation studies, both in the present study and in other studies from our lab have shown a lesser amount of immunogen in the presence of BECC is a better choice than in the absence BECC. The presence of specific cytokines, along with various downstream molecules of T cell activation pathways and CoS, suggests the activation of a slightly Th1-biased adaptive immune response that protects the mice from the onset of Pa infection. These immune molecules and pathways were absent in unimmunized/infected mice, causing those mice to experience a heightened inflammatory response due to the bacterial challenge.

A Th17 response appears to correlate with the protective efficacy of our vaccine platform in mice. While IL-17 downstream signaling tends to give rise to lung inflammation in PBS vaccinated mice, the Th17 pathway in immunized mice tends to be associated with protection the mice. Humans with CF have high levels of IL-17 in their sputum and their airway submucosa is more prone to be infiltrated with Th17 lymphocytes (22, 23). Although this sounds alarming for a vaccine that partly depends on the Th17 response as a component of its efficacy, we have tested our vaccine in CF rats and the results are promising (8–10, 24). Nevertheless, at this time, we do not have results that can confer a precise protective mechanism for our vaccine in these rats against acute Pa infection. With our new vaccine providing broad-spectrum protection in young adult and elderly mice against different serogroups of Pa in pre-clinical studies, this can be an important step toward future clinical studies. Moreover, a more detailed analysis of the immune pathways with the help of single cell transcriptomics may reveal which cells are important behind the positive outcome.

## Data availability statement

The data presented in this study are deposited in the EMBL-EBI repository https://www.ebi.ac.uk/ena with accession number PRJEB74023.

## Ethics statement

All animal studies were carried out in accordance with the IACUC animal use statement (AUS 222-03) from the University of Kansas. The study was conducted in accordance with the local legislation and institutional requirements.

## Author contributions

DH: Conceptualization, Data curation, Formal analysis, Investigation, Methodology, Writing – original draft, Software. RM: Data curation, Methodology, Software, Writing – original draft. TL: Methodology, Writing – review & editing. SM: Methodology, Writing – review & editing. ZD: Methodology, Writing – review & editing. SD: Methodology, Writing – review & editing. SW: Methodology, Writing – review & editing. AN: Funding acquisition, Methodology, Writing – review & editing. SB: Methodology, Writing – review & editing. DV: Methodology, Writing – review & editing. FG: Methodology, Writing – review & editing. RE: Funding acquisition, Investigation, Validation, Writing – review & editing. WDP: Conceptualization, Investigation, Methodology, Supervision, Validation, Writing – review & editing. WLP: Conceptualization, Funding acquisition, Investigation, Project administration, Resources, Supervision, Validation, Writing – review & editing.

## References

[1] Horcajada JP Montero M Oliver A Sorli L Luque S Gomez-Zorrilla S. Epidemiology and treatment of multidrug-resistant and extensively drug-resistant pseudomonas aeruginosa infections. Clin Microbiol Rev. (2019) 32. doi: 10.1128/CMR.00031-19 PMC6730496 31462403

[2] National Center for Emerging and Zoonotic Infectious Diseases (NCEZID) DoH, Quality Promotion (DHQP) CfDCaP. Antibiotic resistance threats in the United States. (2019). doi: 10.15620/cdc:82532

[3] Grimwood K Kyd JM Owen SJ Massa HM Cripps AW. Vaccination against respiratory *Pseudomonas aeruginosa* infection. Hum Vaccin Immunother. (2015) 11:14–20. doi: 10.4161/hv.34296 25483510 PMC4514401

[4] Kollef MH Chastre J Fagon JY Francois B Niederman MS Rello J. Global prospective epidemiologic and surveillance study of ventilator-associated pneumonia due to Pseudomonas aeruginosa. Crit Care Med. (2014) 42:2178–87. doi: 10.1097/CCM.0000000000000510 25054674

[5] Migiyama Y Yanagihara K Kaku N Harada Y Yamada K Nagaoka K. Pseudomonas aeruginosa Bacteremia among Immunocompetent and Immunocompromised Patients: Relation to Initial Antibiotic Therapy and Survival. Jpn J Infect Dis. (2016) 69:91–6. doi: 10.7883/yoken.JJID.2014.573 26073727

[6] Merakou C Schaefers MM Priebe GP. Progress toward the elusive *pseudomonas aeruginosa* vaccine. Surg Infect (Larchmt). (2018) 19:757–68. doi: 10.1089/sur.2018.233 30388058

[7] Baker SM McLachlan JB Morici LA. Immunological considerations in the development of *Pseudomonas aeruginosa* vaccines. Hum Vaccin Immunother. (2020) 16:412–8. doi: 10.1080/21645515.2019.1650999 PMC7062425 31368828

[8] Das S Howlader DR Zheng Q Ratnakaram SSK Whittier SK Lu T. Development of a broadly protective, self-adjuvanting subunit vaccine to prevent infections by pseudomonas aeruginosa. Front Immunol. (2020) 11:583008. doi: 10.3389/fimmu.2020.583008 33281815 PMC7705240

[9] Howlader DR Das S Lu T Hu G Varisco DJ Dietz ZK. Effect of two unique nanoparticle formulations on the efficacy of a broadly protective vaccine against pseudomonas aeruginosa. Front Pharmacol. (2021) 12:706157. doi: 10.3389/fphar.2021.706157 34483911 PMC8416447

[10] Howlader DR Das S Lu T Mandal RS Hu G Varisco DJ. A protein subunit vaccine elicits a balanced immune response that protects against *Pseudomonas* pulmonary infection. NPJ Vaccines. (2023) 8:37. doi: 10.1038/s41541-023-00618-w 36918600 PMC10012293

[11] Lu T Das S Howlader DR Zheng Q Siva Sai Kumar R Whittier SK. L-DBF elicits cross protection against different serotypes of Shigella spp. Front Trop Dis. (2021) 2. doi: 10.3389/fitd.2021.729731

[12] Priebe GP Goldberg JB. Vaccines for *Pseudomonas aeruginosa*: a long and winding road. Expert Rev Vaccines. (2014) 13:507–19. doi: 10.1586/14760584.2014.890053 PMC4521563 24575895

[13] Huber P. ExlA: A new contributor to pseudomonas aeruginosa virulence. Front Cell Infect Microbiol. (2022) 12:929150. doi: 10.3389/fcimb.2022.929150 35811671 PMC9260685

[14] Thrane SW Taylor VL Freschi L Kukavica-Ibrulj I Boyle B Laroche J. The Widespread Multidrug-Resistant Serotype O12 Pseudomonas aeruginosa Clone Emerged through Concomitant Horizontal Transfer of Serotype Antigen and Antibiotic Resistance Gene Clusters. mBio. (2015) 6:e01396–15. doi: 10.1128/mBio.01396-15 PMC4600120 26396243

[15] Lavoie EG Wangdi T Kazmierczak BI. Innate immune responses to Pseudomonas aeruginosa infection. Microbes Infect. (2011) 13:1133–45. doi: 10.1016/j.micinf.2011.07.011 PMC3221798 21839853

[16] Jelsbak L Johansen HK Frost AL Thogersen R Thomsen LE Ciofu O. Molecular epidemiology and dynamics of Pseudomonas aeruginosa populations in lungs of cystic fibrosis patients. Infect Immun. (2007) 75:2214–24. doi: 10.1128/IAI.01282-06 PMC1865789 17261614

[17] Ge SX Son EW Yao R. iDEP: an integrated web application for differential expression and pathway analysis of RNA-Seq data. BMC Bioinf. (2018) 19:534. doi: 10.1186/s12859-018-2486-6 PMC6299935 30567491

[18] National Center for Health Statistics (US). Health, United States, 2019. Hyattsville (MD): National Center for Health Statistics (US); (2021). doi: 10.15620/cdc:100685 33818995

[19] Montecino-Rodriguez E Berent-Maoz B Dorshkind K. Causes, consequences, and reversal of immune system aging. J Clin Invest. (2013) 123:958–65. doi: 10.1172/JCI64096 PMC3582124 23454758

[20] Hu G Varisco DJ Das S Middaugh CR Gardner F Ernst RK. Physicochemical characterization of biological and synthetic forms of two lipid A-based TLR4 agonists. Heliyon. (2023) 9:e18119. doi: 10.1016/j.heliyon.2023.e18119 37483830 PMC10362264

[21] Holmgren J Czerkinsky C. Mucosal immunity and vaccines. Nat Med. (2005) 11:S45–53. doi: 10.1038/nm1213 15812489

[22] Bretscher P. On analyzing how the Th1/Th2 phenotype of an immune response is determined: classical observations must not be ignored. Front Immunol. (2019) 10:1234. doi: 10.3389/fimmu.2019.01234 31231378 PMC6560152

[23] Wu W Huang J Duan B Traficante DC Hong H Risech M. Th17-stimulating protein vaccines confer protection against *Pseudomonas aeruginosa* pneumonia. Am J Respir Crit Care Med. (2012) 186:420–7. doi: 10.1164/rccm.201202-0182OC PMC3443805 22723292

[24] Das S Howlader DR Lu T Whittier SK Hu G Sharma S. Immunogenicity and protective efficacy of nanoparticle formulations of L-SseB against Salmonella infection. Front Immunol. (2023) 14:1208848. doi: 10.3389/fimmu.2023.1208848 37457702 PMC10347375

