# Supplementary material for: Development of a nano-emulsion based multivalent protein subunit vaccine against Pseudomonas aeruginosa
Source: Front Immunol. 2024 Apr 18;15:1372349. doi: 10.3389/fimmu.2024.1372349 (PMC11063228; doi:10.3389/fimmu.2024.1372349)
Supplement: Supplementary file 1 [file DataSheet_1.pdf]

Development of a nano-emulsion based multivalent protein subunit vaccine against *Pseudomonas aeruginosa*

Debaki R Howlader[1,2,3], Rahul Shubhra Mandal[5], Ti Lu[1,2,3], Suhrid Maiti[1,2], Zackary K Dietz[1,2,], Sayan Das[3,6], Sean K Whittier[1,2,3], Aaron C. Nagel[4], Satabdi Biswas[1,2], David J Varisco[6], Francesca M Gardner[6], Robert K Ernst[6], William D Picking[1,2,3], and Wendy L Picking[1,2,3,4*]

[1]Department of Veterinary Pathobiology, Center for Veterinary Medicine, and [2]Bond Life Science Center, University of Missouri, Columbia, Missouri 65211 and [3]Department of Pharmaceutical Chemistry, University of Kansas, Lawrence, KS 66047, [4]Hafion, Inc., Lawrence, KS 66047, [5] Perelman School of Medicine, University of Pennsylvania, Philadelphia, PA, 19104, USA, [6]Department of Microbial Pathogenesis, University of Maryland, Baltimore, MD 21201.

*Corresponding Author:

Wendy L Picking, wendy.picking@missouri.edu

Supplemental Figure 1.

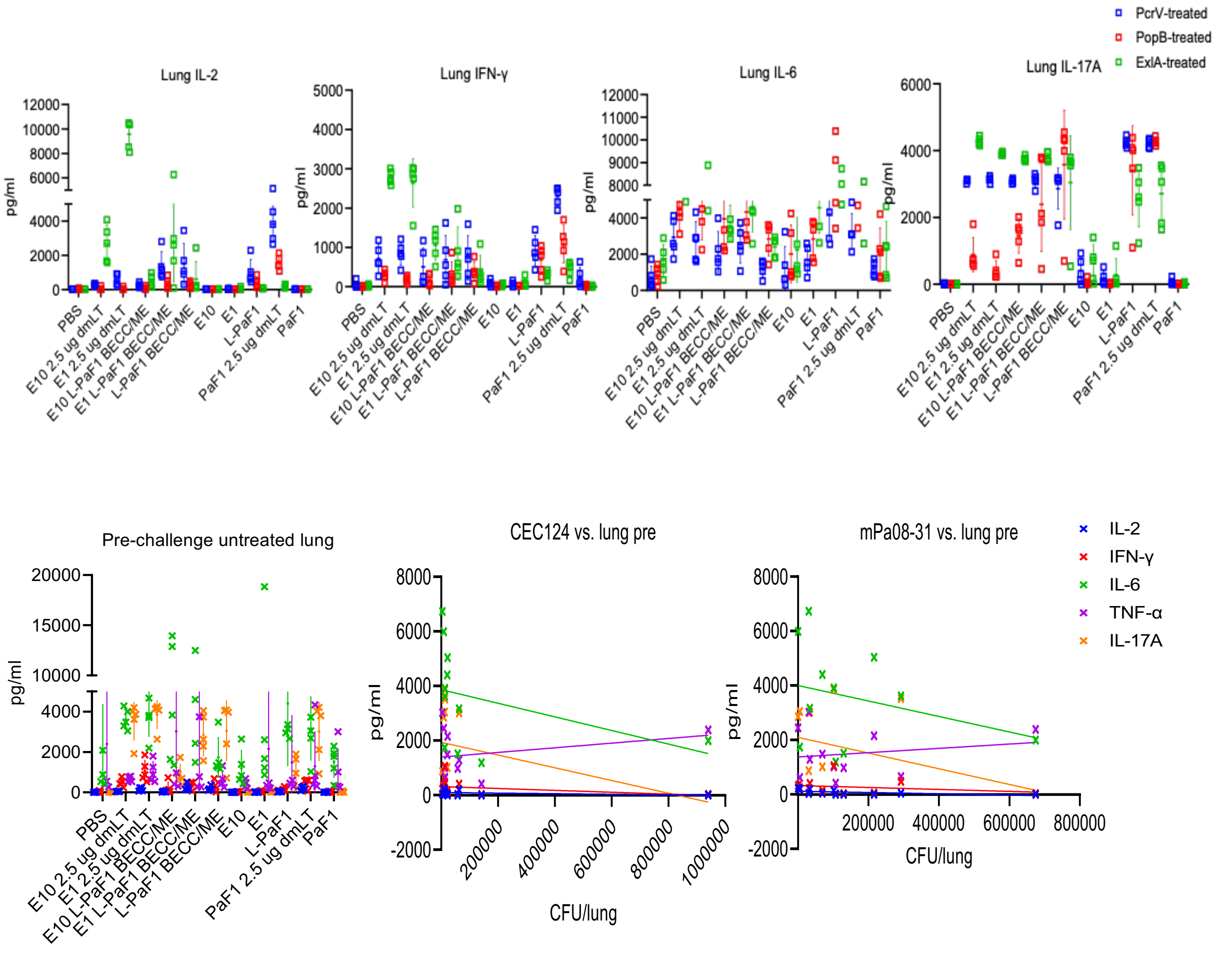

Supplemental Figure 1. Pre-challenge lung cytokines. Single cell lung suspensions were prepared as described in the text. Lung cells were incubated at 37° C, for 48 h without any protein stimulation. The top panel shows different lung cytokines following stimulation with PcrV or PopB or ExlA. The bottom panel shows same cytokines but in the absence of protein stimulation. Supplemental table 1 shows the precise values of the top panels, whereas the values associated to the bottom panels can be found in Suppemental table 4. The bottom middle and right panels show the correlation between lung burden (CEC124 and mPa08-31, respectively) and pre-challenge cytokines. Values were plotted as individual points ± SD (n = 5/group). Error bars represent SD. Both 'r' and simple linear regression were calculated at 95% confidence level.

Supplemental Figure 2.

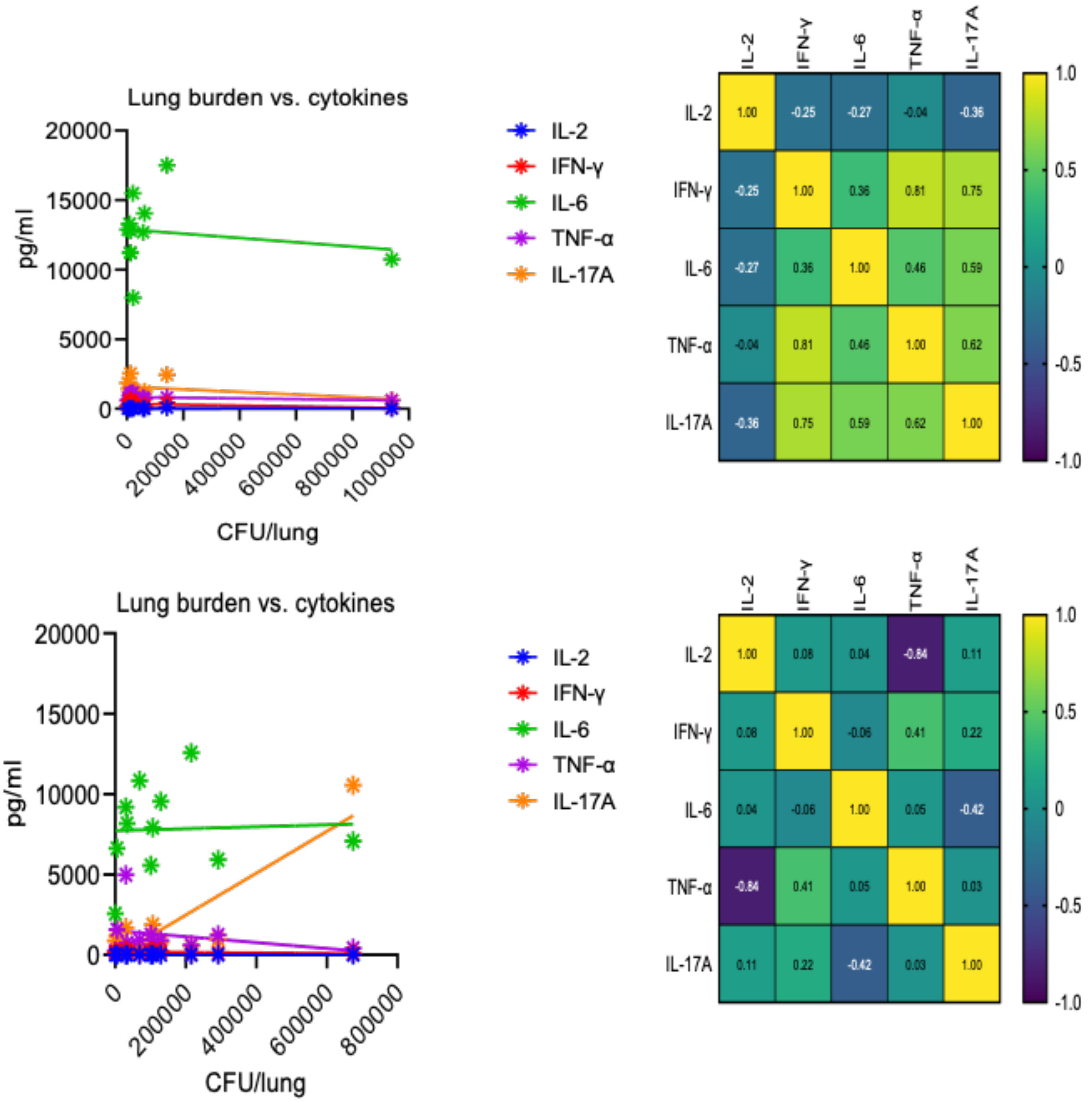

Supplemental Figure 2. Post-challenge lung cytokines and correlations with CFU burden. Correlation between lung burden and post-challenge cytokines were measured for CED124 (**top left panel**) and mPa08-31 (**bottom left panel**) challenged mice. Values of Spearman's r is shown in the corresponding heat maps (**top right panel** and **bottom right panel**). Both 'r' and simple linear regression were calculated at 95% confidence level. *$p < 0.05$, **$p < 0.01$, ***$p < 0.001$, ****$p < 0.0001$.

Supplemental Figure 3A

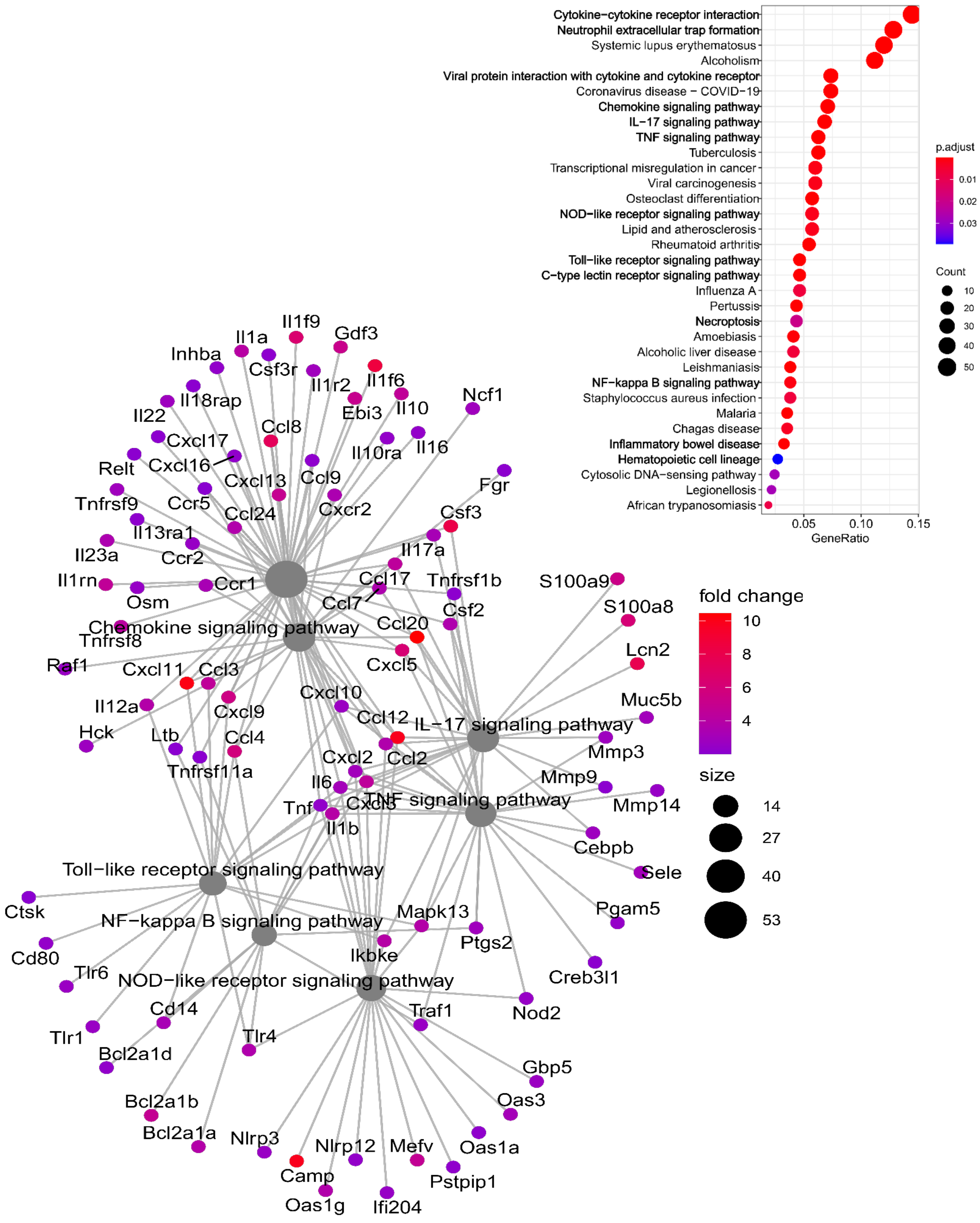

Supplemental Figure 3B

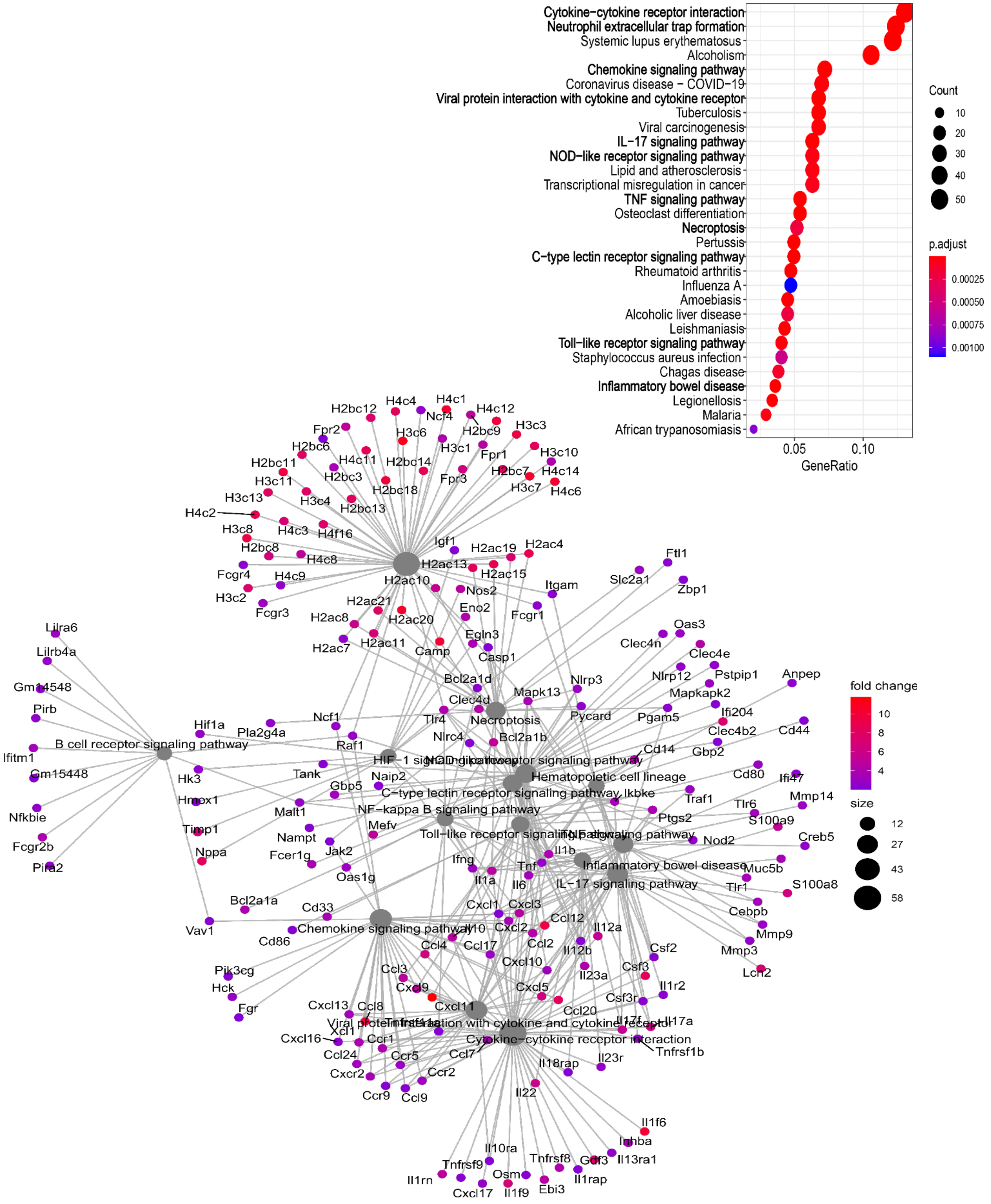

Supplemental Figure 3C

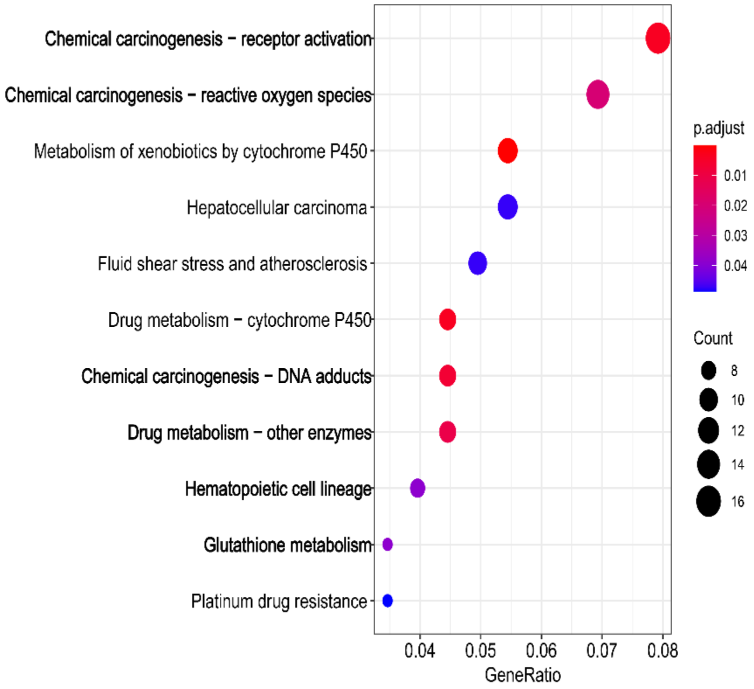

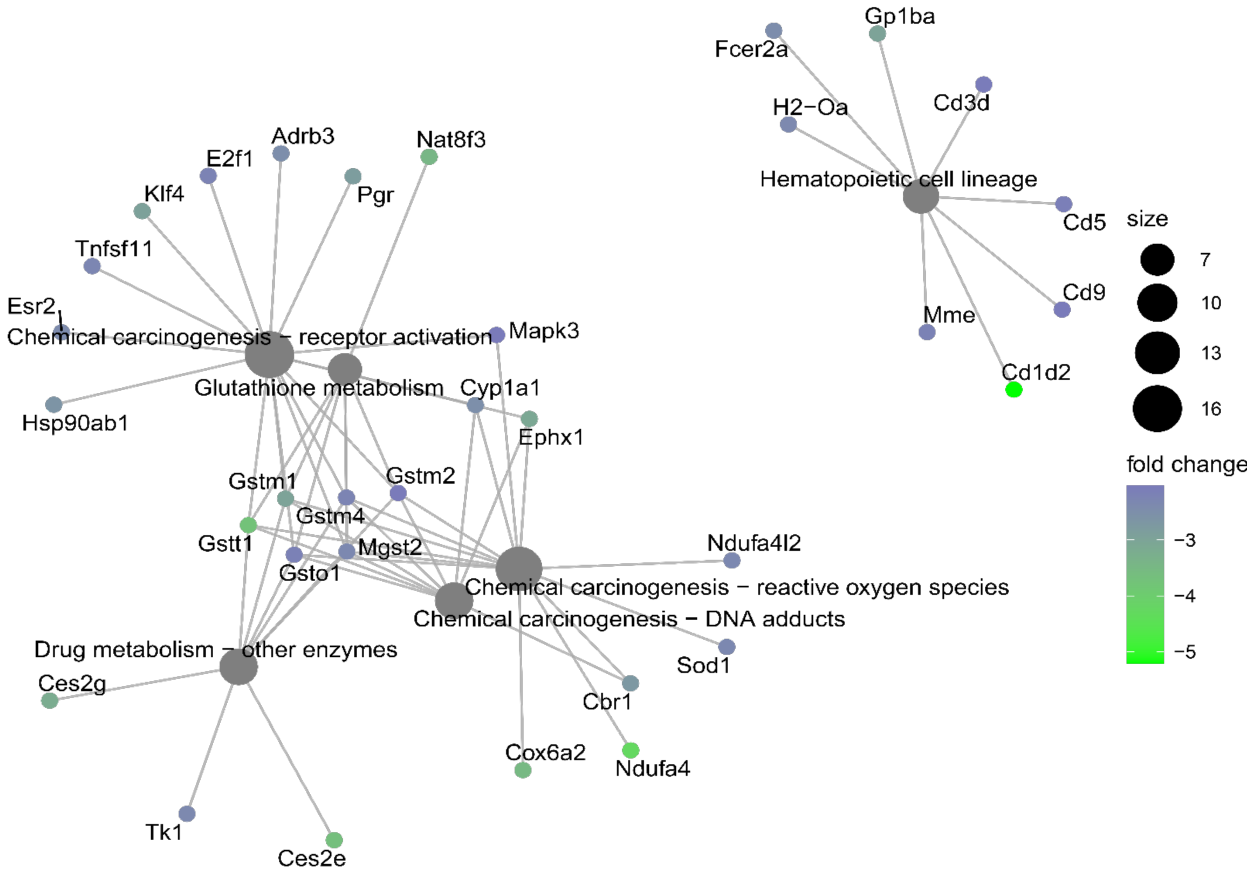

Supplemental Figure 3D

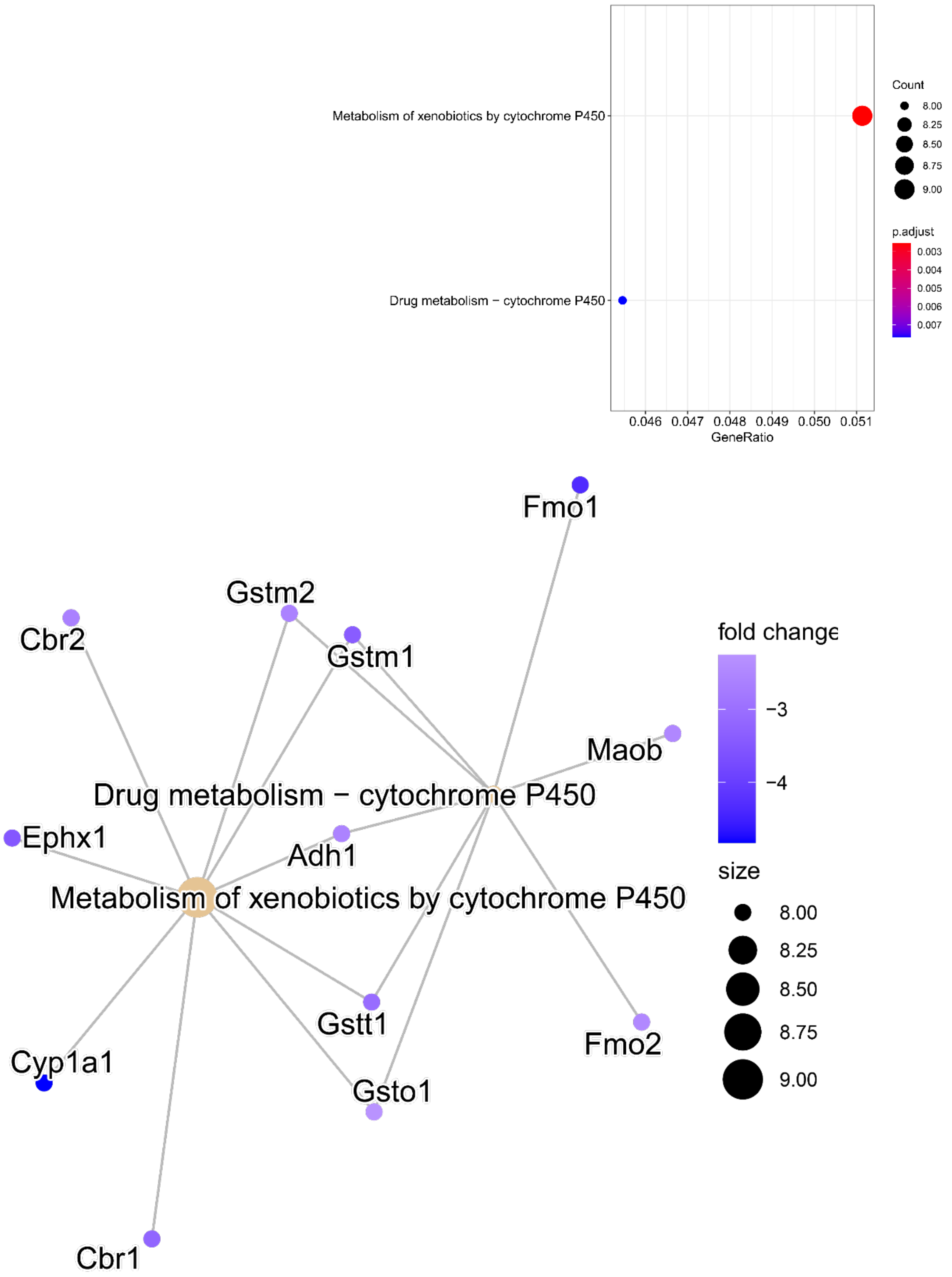

Supplemental Figure 3: Up- and down-regulated pathways in CEC124, and mPa08-31 infected mice. Naïve elderly BALB/c mice were infected with either CEC124, or mPa08-31 as described in the text to determine the similarities and/or differences between them. **A and C** show the up and downregulated pathways, respectively, in elderly mice infected with CEC124. **B and D** show the up and downregulated pathways, respectively, in elderly mice infected with mPa08-31.

Supplemental Figure 4.

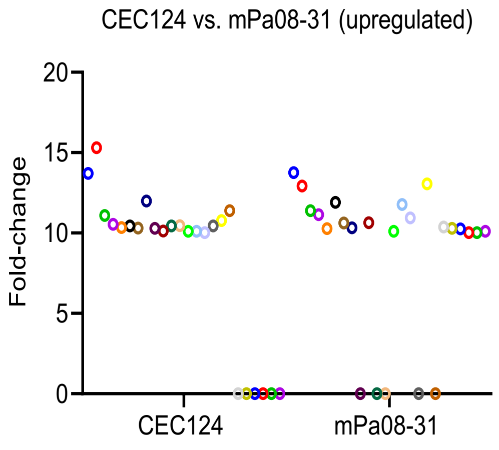

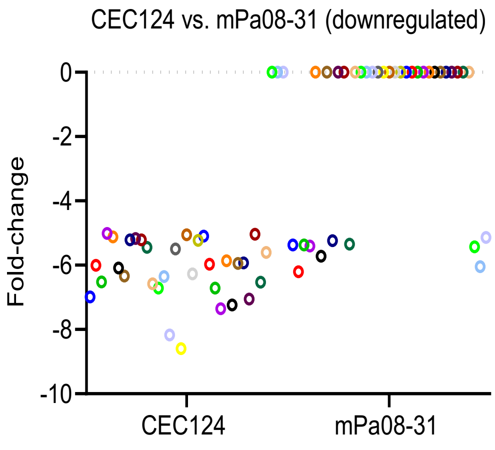

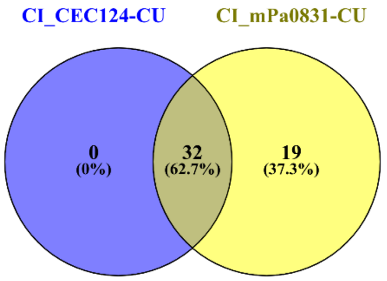

Supplemental Figure 4. Unique genes, and common pathways involved to set in Pa infection. Top panel. Genes with more than equal 10-fold upregulation, and less than equal 5-fold downregulation were taken into consideration post-infection. **Bottom panel.** Common pathways involved in the course of infection in elderly mice.

Supplemental Figure 5.

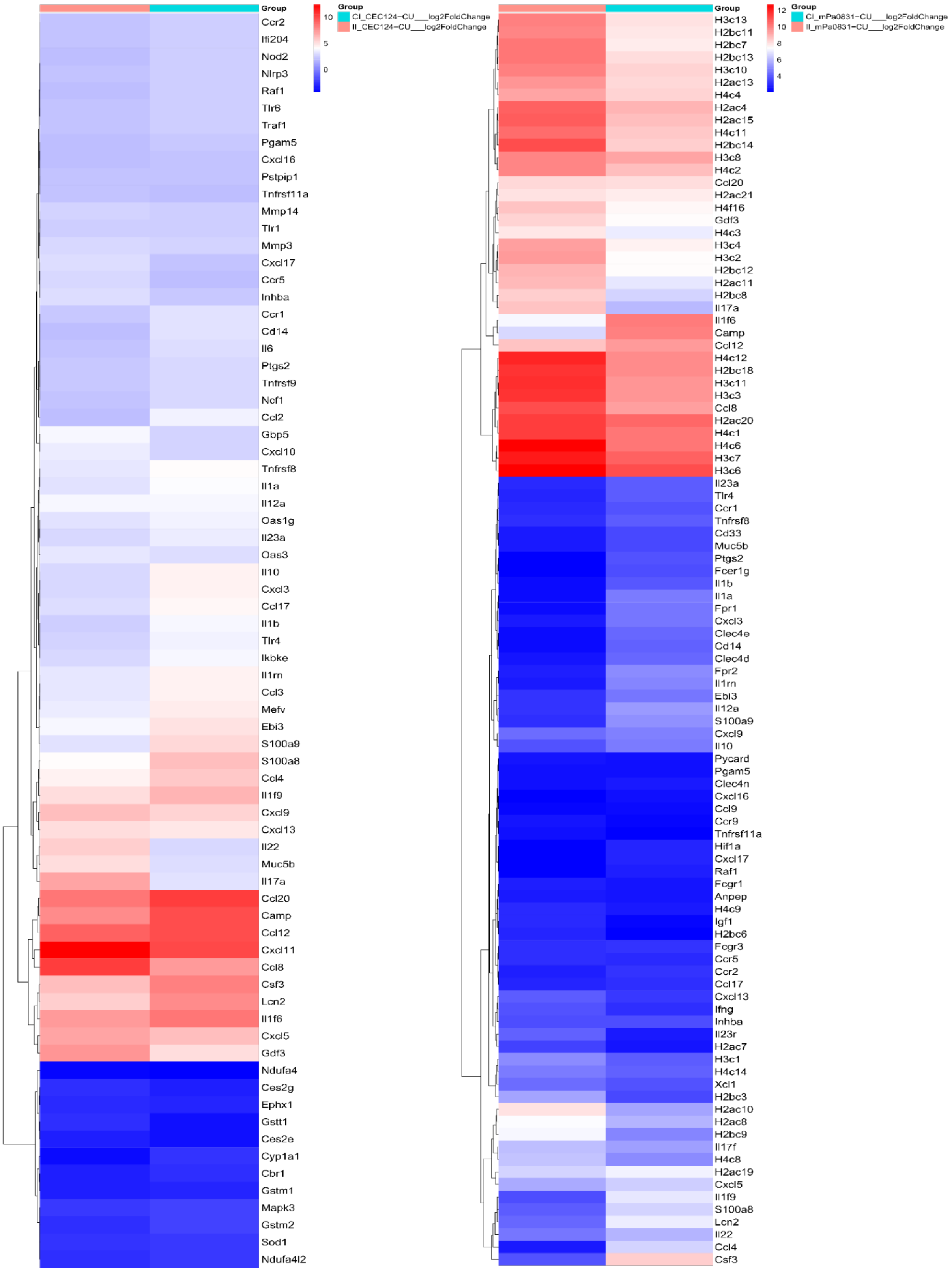

Supplemental Figure 5. Heatmaps showing similarities and differences in gene profile of control and immunized mice. The **left** panel shows the similarities and differences in gene profile in CEC124 infected control, and immunized elderly mice. The **right** panel shows the similarities and differences in gene profile in mPa08-31 infected control, and immunized elderly mice.

Supplemental Figure 6.

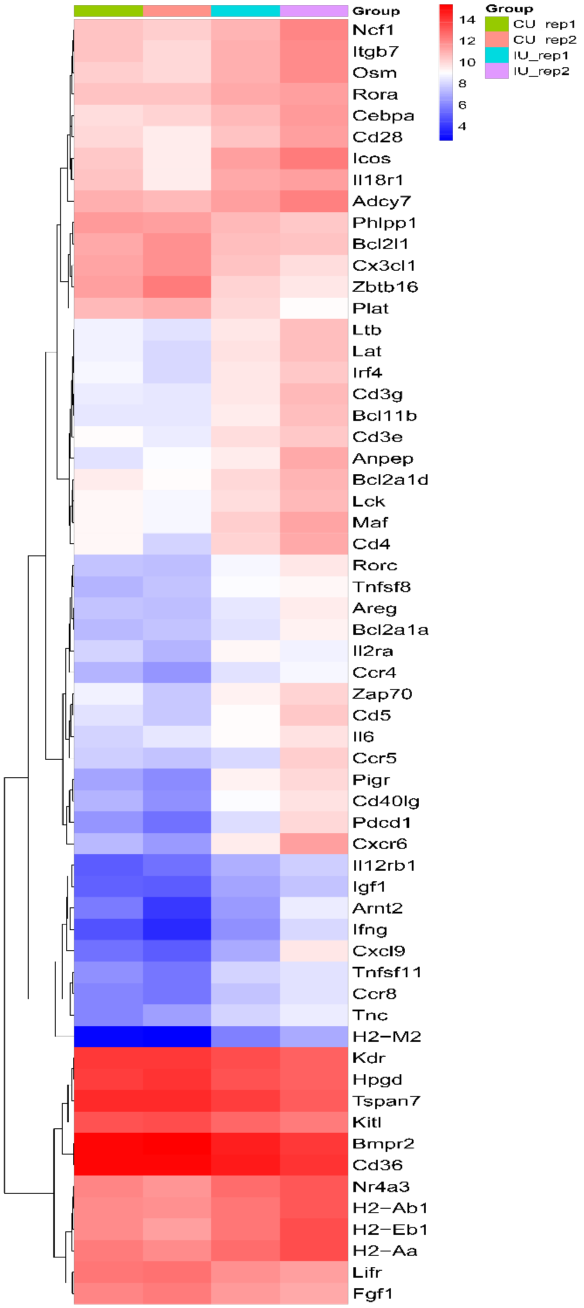

Supplemental Figure 6. Heatmap showing the similarities and differences in gene profile before, and after immunization. This is an extended version of the heatmap shown on Figure 4. See text for details.
