# Supplementary material for: Development of a nano-emulsion based multivalent protein subunit vaccine against Pseudomonas aeruginosa
Source: Front Immunol. 2024 Apr 18;15:1372349. doi: 10.3389/fimmu.2024.1372349 (PMC11063228; doi:10.3389/fimmu.2024.1372349)
Supplement: Supplementary file 2 [file DataSheet_2.pdf]

Development of a nano-emulsion based multivalent protein subunit vaccine against *Pseudomonas aeruginosa*

Debaki R Howlader[1,2,3], Rahul Shubhra Mandal[5], Ti Lu[1,2,3], Suhrid Maiti[1,2], Zackary K Dietz[1,2,], Sayan Das[3,6], Sean K Whittier[1,2,3], Aaron C. Nagel[4], Satabdi Biswas[1,2], David J Varisco[6], Francesca M Gardner[6], Robert K Ernst[6], William D Picking[1,2,3], and Wendy L Picking[1,2,3,4*]

[1]Department of Veterinary Pathobiology, Center for Veterinary Medicine, and [2]Bond Life Science Center, University of Missouri, Columbia, Missouri 65211 and [3]Department of Pharmaceutical Chemistry, University of Kansas, Lawrence, KS 66047, [4]Hafion, Inc., Lawrence, KS 66047, [5] Perelman School of Medicine, University of Pennsylvania, Philadelphia, PA, 19104, USA, [6]Department of Microbial Pathogenesis, University of Maryland, Baltimore, MD 21201.

*Corresponding Author:

Wendy L Picking, wendy.picking@missouri.edu

Supplemental Table 1: ELISpot:

A. PcrV-treated pre-challenge lung IFN-γ:

Dunn's multiple comparisons test	Mean rank diff.	Significant?	Summary	Adjusted P Value
PBS vs. E10 2.5 ug dmLT	-33.10	Yes	*	0.0107
PBS vs. E1 2.5 ug dmLT	-36.30	Yes	**	0.0034
PBS vs. E10 L-PaF1 BECC/ME	-24.50	No	ns	0.1550
PBS vs. E1 L-PaF1 BECC/ME	-28.90	Yes	*	0.0430
PBS vs. L-PaF1 BECC/ME	-22.60	No	ns	0.2556
PBS vs. E10	-1.800	No	ns	>0.9999
PBS vs. E1	-6.300	No	ns	>0.9999
PBS vs. L-PaF1	-29.50	Yes	*	0.0356
PBS vs. PaF1 2.5 ug dmLT	-36.30	Yes	**	0.0034
PBS vs. PaF1	-4.000	No	ns	>0.9999

B. PopB-treated pre-challenge lung IFN-γ:

Dunn's multiple comparisons test	Mean rank diff.	Significant?	Summary	Adjusted P Value
PBS vs. E10 2.5 ug dmLT	-27.40	No	ns	0.0660
PBS vs. E1 2.5 ug dmLT	-27.10	No	ns	0.0722
PBS vs. E10 L-PaF1 BECC/ME	-12.10	No	ns	>0.9999
PBS vs. E1 L-PaF1 BECC/ME	-29.70	Yes	*	0.0324
PBS vs. L-PaF1 BECC/ME	-18.20	No	ns	0.7119
PBS vs. E10	-0.5000	No	ns	>0.9999
PBS vs. E1	-6.800	No	ns	>0.9999
PBS vs. L-PaF1	-33.70	Yes	**	0.0084
PBS vs. PaF1 2.5 ug dmLT	-37.10	Yes	**	0.0024
PBS vs. PaF1	0.1000	No	ns	>0.9999

C. ExlA-treated pre-challenge lung IFN-γ:

Dunn's multiple comparisons test	Mean rank diff.	Significant?	Summary	Adjusted P Value
PBS vs. E10 2.5 ug dmLT	-38.80	Yes	**	0.0013
PBS vs. E1 2.5 ug dmLT	-24.00	No	ns	0.1778
PBS vs. E10 L-PaF1 BECC/ME	-33.80	Yes	**	0.0084
PBS vs. E1 L-PaF1 BECC/ME	-30.60	Yes	*	0.0251
PBS vs. L-PaF1 BECC/ME	-14.30	No	ns	>0.9999
PBS vs. E10	-2.600	No	ns	>0.9999
PBS vs. E1	-3.400	No	ns	>0.9999
PBS vs. L-PaF1	-17.60	No	ns	0.8220
PBS vs. PaF1 2.5 ug dmLT	-16.60	No	ns	>0.9999
PBS vs. PaF1	3.500	No	ns	>0.9999

D. Untreated pre-challenge lung IFN-γ:

Dunn's multiple comparisons test	Mean rank diff.	Significant?	Summary	Adjusted P Value
PBS vs. E10 2.5 ug dmLT	-20.80	No	ns	0.3951
PBS vs. E1 2.5 ug dmLT	-27.40	No	ns	0.0668
PBS vs. E10 L-PaF1 BECC/ME	-9.700	No	ns	>0.9999
PBS vs. E1 L-PaF1 BECC/ME	-7.500	No	ns	>0.9999
PBS vs. L-PaF1 BECC/ME	5.800	No	ns	>0.9999
PBS vs. E10	13.50	No	ns	>0.9999
PBS vs. E1	11.40	No	ns	>0.9999
PBS vs. L-PaF1	-1.200	No	ns	>0.9999
PBS vs. PaF1 2.5 ug dmLT	-6.900	No	ns	>0.9999
PBS vs. PaF1	16.40	No	ns	>0.9999

E. PcrV-treated pre-challenge lung IL-17A:

Dunn's multiple comparisons test	Mean rank diff.	Significant?	Summary	Adjusted P Value
PBS vs. E10 2.5 ug dmLT	-24.10	No	ns	0.1736
PBS vs. E1 2.5 ug dmLT	-25.70	No	ns	0.1118
PBS vs. E10 L-PaF1 BECC/ME	-39.40	Yes	**	0.0010
PBS vs. E1 L-PaF1 BECC/ME	-35.90	Yes	**	0.0039
PBS vs. L-PaF1 BECC/ME	-34.90	Yes	**	0.0057
PBS vs. E10	-12.10	No	ns	>0.9999
PBS vs. E1	-11.30	No	ns	>0.9999
PBS vs. L-PaF1	-41.00	Yes	***	0.0005
PBS vs. PaF1 2.5 ug dmLT	-23.80	No	ns	0.1880
PBS vs. PaF1	-5.900	No	ns	>0.9999

F. PopB-treated pre-challenge lung IL-17A:

Dunn's multiple comparisons test	Mean rank diff.	Significant?	Summary	Adjusted P Value
PBS vs. E10 2.5 ug dmLT	-29.80	Yes	*	0.0326
PBS vs. E1 2.5 ug dmLT	-35.80	Yes	**	0.0041
PBS vs. E10 L-PaF1 BECC/ME	-26.60	No	ns	0.0864
PBS vs. E1 L-PaF1 BECC/ME	-29.60	Yes	*	0.0348
PBS vs. L-PaF1 BECC/ME	-28.00	No	ns	0.0571
PBS vs. E10	-4.700	No	ns	>0.9999
PBS vs. E1	-6.500	No	ns	>0.9999
PBS vs. L-PaF1	-40.60	Yes	***	0.0006
PBS vs. PaF1 2.5 ug dmLT	-30.80	Yes	*	0.0236
PBS vs. PaF1	-5.200	No	ns	>0.9999

G. ExlA-treated pre-challenge lung IL-17A:

Dunn's multiple comparisons test	Mean rank diff.	Significant?	Summary	Adjusted P Value
PBS vs. E10 2.5 ug dmLT	1.200	No	ns	>0.9999
PBS vs. E1 2.5 ug dmLT	-0.1000	No	ns	>0.9999
PBS vs. E10 L-PaF1 BECC/ME	-27.90	No	ns	0.0589
PBS vs. E1 L-PaF1 BECC/ME	-29.90	Yes	*	0.0316
PBS vs. L-PaF1 BECC/ME	-28.50	Yes	*	0.0490
PBS vs. E10	-19.30	No	ns	0.5677
PBS vs. E1	-15.70	No	ns	>0.9999
PBS vs. L-PaF1	-36.70	Yes	**	0.0029
PBS vs. PaF1 2.5 ug dmLT	-28.70	Yes	*	0.0461
PBS vs. PaF1	-4.700	No	ns	>0.9999

H. Untreated pre-challenge lung IL-17A:

Dunn's multiple comparisons test	Mean rank diff.	Significant?	Summary	Adjusted P Value
PBS vs. E10 2.5 ug dmLT	-38.30	Yes	**	0.0016
PBS vs. E1 2.5 ug dmLT	-13.70	No	ns	>0.9999
PBS vs. E10 L-PaF1 BECC/ME	-21.10	No	ns	0.3729
PBS vs. E1 L-PaF1 BECC/ME	-29.30	Yes	*	0.0383
PBS vs. L-PaF1 BECC/ME	-30.90	Yes	*	0.0229
PBS vs. E10	0.1000	No	ns	>0.9999
PBS vs. E1	-0.9000	No	ns	>0.9999
PBS vs. L-PaF1	-23.10	No	ns	0.2261
PBS vs. PaF1 2.5 ug dmLT	-19.50	No	ns	0.5427
PBS vs. PaF1	4.000	No	ns	>0.9999

Supplemental Table 1. Statistical analyses of ELISpot assay from pre-challenge young mice. See text for details.

Supplemental Table 2: MSD:

A. PcrV-treated pre-challenge lung IL-2:

Dunn's multiple comparisons test	Mean rank diff.	Significant?	Summary	Adjusted P Value
PBS vs. E10 2.5 ug dmLT	-23.40	No	ns	0.2092
PBS vs. E1 2.5 ug dmLT	-29.80	Yes	*	0.0327
PBS vs. E10 L-PaF1 BECC/ME	-20.60	No	ns	0.4205
PBS vs. E1 L-PaF1 BECC/ME	-38.80	Yes	**	0.0013
PBS vs. L-PaF1 BECC/ME	-37.40	Yes	**	0.0022
PBS vs. E10	-7.800	No	ns	>0.9999
PBS vs. E1	-8.800	No	ns	>0.9999
PBS vs. L-PaF1	-34.80	Yes	**	0.0059
PBS vs. PaF1 2.5 ug dmLT	-47.60	Yes	****	<0.0001
PBS vs. PaF1	-6.200	No	ns	>0.9999

B. PopB-treated pre-challenge lung IL-2:

Dunn's multiple comparisons test	Mean rank diff.	Significant?	Summary	Adjusted P Value
PBS vs. E10 2.5 ug dmLT	-18.00	No	ns	0.7566
PBS vs. E1 2.5 ug dmLT	-13.40	No	ns	>0.9999
PBS vs. E10 L-PaF1 BECC/ME	-22.40	No	ns	0.2706
PBS vs. E1 L-PaF1 BECC/ME	-32.40	Yes	*	0.0139
PBS vs. L-PaF1 BECC/ME	-30.80	Yes	*	0.0237
PBS vs. E10	-3.200	No	ns	>0.9999
PBS vs. E1	-1.600	No	ns	>0.9999
PBS vs. L-PaF1	-34.80	Yes	**	0.0059
PBS vs. PaF1 2.5 ug dmLT	-43.80	Yes	***	0.0002
PBS vs. PaF1	-6.400	No	ns	>0.9999

C. ExlA-treated pre-challenge lung IL-2:

Dunn's multiple comparisons test	Mean rank diff.	Significant?	Summary	Adjusted P Value
PBS vs. E10 2.5 ug dmLT	-38.80	Yes	**	0.0013
PBS vs. E1 2.5 ug dmLT	-46.60	Yes	****	<0.0001
PBS vs. E10 L-PaF1 BECC/ME	-29.80	Yes	*	0.0327
PBS vs. E1 L-PaF1 BECC/ME	-35.20	Yes	**	0.0051
PBS vs. L-PaF1 BECC/ME	-26.60	No	ns	0.0866
PBS vs. E10	-8.600	No	ns	>0.9999
PBS vs. E1	-11.00	No	ns	>0.9999
PBS vs. L-PaF1	-16.00	No	ns	>0.9999
PBS vs. PaF1 2.5 ug dmLT	-25.60	No	ns	0.1152
PBS vs. PaF1	0.6000	No	ns	>0.9999

D. PcrV-treated pre-challenge lung IFN-γ:

Dunn's multiple comparisons test	Mean rank diff.	Significant?	Summary	Adjusted P Value
PBS vs. E10 2.5 ug dmLT	-26.80	No	ns	0.0817
PBS vs. E1 2.5 ug dmLT	-30.20	Yes	*	0.0288
PBS vs. E10 L-PaF1 BECC/ME	-19.80	No	ns	0.5069
PBS vs. E1 L-PaF1 BECC/ME	-23.20	No	ns	0.2204
PBS vs. L-PaF1 BECC/ME	-25.60	No	ns	0.1152
PBS vs. E10	-4.400	No	ns	>0.9999
PBS vs. E1	-3.200	No	ns	>0.9999
PBS vs. L-PaF1	-31.00	Yes	*	0.0222
PBS vs. PaF1 2.5 ug dmLT	-45.00	Yes	****	<0.0001
PBS vs. PaF1	-10.80	No	ns	>0.9999

E. PopB-treated pre-challenge lung IFN-γ:

Dunn's multiple comparisons test	Mean rank diff.	Significant?	Summary	Adjusted P Value
PBS vs. E10 2.5 ug dmLT	-31.20	Yes	*	0.0208
PBS vs. E1 2.5 ug dmLT	-25.00	No	ns	0.1361
PBS vs. E10 L-PaF1 BECC/ME	-23.80	No	ns	0.1883
PBS vs. E1 L-PaF1 BECC/ME	-29.80	Yes	*	0.0327
PBS vs. L-PaF1 BECC/ME	-33.60	Yes	**	0.0091
PBS vs. E10	-6.400	No	ns	>0.9999
PBS vs. E1	-8.200	No	ns	>0.9999
PBS vs. L-PaF1	-40.60	Yes	***	0.0006
PBS vs. PaF1 2.5 ug dmLT	-46.40	Yes	****	<0.0001
PBS vs. PaF1	-8.000	No	ns	>0.9999

F. ExlA-treated pre-challenge lung IFN-γ:

Dunn's multiple comparisons test	Mean rank diff.	Significant?	Summary	Adjusted P Value
PBS vs. E10 2.5 ug dmLT	-42.00	Yes	***	0.0003
PBS vs. E1 2.5 ug dmLT	-42.80	Yes	***	0.0002
PBS vs. E10 L-PaF1 BECC/ME	-32.40	Yes	*	0.0139
PBS vs. E1 L-PaF1 BECC/ME	-27.80	No	ns	0.0608
PBS vs. L-PaF1 BECC/ME	-20.00	No	ns	0.4840
PBS vs. E10	-4.600	No	ns	>0.9999
PBS vs. E1	-8.800	No	ns	>0.9999
PBS vs. L-PaF1	-19.20	No	ns	0.5811
PBS vs. PaF1 2.5 ug dmLT	-24.40	No	ns	0.1604
PBS vs. PaF1	2.000	No	ns	>0.9999

G. PcrV-treated pre-challenge lung IL-6:

Dunn's multiple comparisons test	Mean rank diff.	Significant?	Summary	Adjusted P Value
PBS vs. E10 2.5 ug dmLT	-30.60	Yes	*	0.0253
PBS vs. E1 2.5 ug dmLT	-27.40	No	ns	0.0685
PBS vs. E10 L-PaF1 BECC/ME	-19.80	No	ns	0.5069
PBS vs. E1 L-PaF1 BECC/ME	-25.40	No	ns	0.1218
PBS vs. L-PaF1 BECC/ME	-7.600	No	ns	>0.9999
PBS vs. E10	-7.000	No	ns	>0.9999
PBS vs. E1	-14.20	No	ns	>0.9999
PBS vs. L-PaF1	-38.20	Yes	**	0.0016
PBS vs. PaF1 2.5 ug dmLT	-33.60	Yes	**	0.0091
PBS vs. PaF1	-7.400	No	ns	>0.9999

H. PopB-treated pre-challenge lung IL-6:

Dunn's multiple comparisons test	Mean rank diff.	Significant?	Summary	Adjusted P Value
PBS vs. E10 2.5 ug dmLT	-31.20	Yes	*	0.0208
PBS vs. E1 2.5 ug dmLT	-31.40	Yes	*	0.0194
PBS vs. E10 L-PaF1 BECC/ME	-24.80	No	ns	0.1438
PBS vs. E1 L-PaF1 BECC/ME	-27.60	No	ns	0.0645
PBS vs. L-PaF1 BECC/ME	-16.60	No	ns	>0.9999
PBS vs. E10	-9.800	No	ns	>0.9999
PBS vs. E1	-17.20	No	ns	0.8960
PBS vs. L-PaF1	-40.20	Yes	***	0.0007
PBS vs. PaF1 2.5 ug dmLT	-36.20	Yes	**	0.0035
PBS vs. PaF1	-9.200	No	ns	>0.9999

I. ExlA-treated pre-challenge lung IL-6:

Dunn's multiple comparisons test	Mean rank diff.	Significant?	Summary	Adjusted P Value
PBS vs. E10 2.5 ug dmLT	-30.80	Yes	*	0.0237
PBS vs. E1 2.5 ug dmLT	-36.00	Yes	**	0.0038
PBS vs. E10 L-PaF1 BECC/ME	-16.80	No	ns	0.9731
PBS vs. E1 L-PaF1 BECC/ME	-22.60	No	ns	0.2572
PBS vs. L-PaF1 BECC/ME	-6.400	No	ns	>0.9999
PBS vs. E10	-4.000	No	ns	>0.9999
PBS vs. E1	-23.00	No	ns	0.2321
PBS vs. L-PaF1	-36.40	Yes	**	0.0033
PBS vs. PaF1 2.5 ug dmLT	-28.00	No	ns	0.0572
PBS vs. PaF1	-2.800	No	ns	>0.9999

J. PcrV-treated pre-challenge lung IL-17A:

Dunn's multiple comparisons test	Mean rank diff.	Significant?	Summary	Adjusted P Value
PBS vs. E10 2.5 ug dmLT	-26.60	No	ns	0.0866
PBS vs. E1 2.5 ug dmLT	-31.00	Yes	*	0.0222
PBS vs. E10 L-PaF1 BECC/ME	-25.00	No	ns	0.1361
PBS vs. E1 L-PaF1 BECC/ME	-29.00	Yes	*	0.0421
PBS vs. L-PaF1 BECC/ME	-27.40	No	ns	0.0685
PBS vs. E10	-9.600	No	ns	>0.9999
PBS vs. E1	-7.400	No	ns	>0.9999
PBS vs. L-PaF1	-45.40	Yes	****	<0.0001
PBS vs. PaF1 2.5 ug dmLT	-45.20	Yes	****	<0.0001
PBS vs. PaF1	-4.200	No	ns	>0.9999

K. PopB-treated pre-challenge lung IL-17A:

Dunn's multiple comparisons test	Mean rank diff.	Significant?	Summary	Adjusted P Value
PBS vs. E10 2.5 ug dmLT	-26.20	No	ns	0.0972
PBS vs. E1 2.5 ug dmLT	-20.20	No	ns	0.4620
PBS vs. E10 L-PaF1 BECC/ME	-30.40	Yes	*	0.0270
PBS vs. E1 L-PaF1 BECC/ME	-33.60	Yes	**	0.0091
PBS vs. L-PaF1 BECC/ME	-42.20	Yes	***	0.0003
PBS vs. E10	-10.80	No	ns	>0.9999
PBS vs. E1	-8.400	No	ns	>0.9999
PBS vs. L-PaF1	-39.60	Yes	***	0.0009
PBS vs. PaF1 2.5 ug dmLT	-45.60	Yes	****	<0.0001
PBS vs. PaF1	-7.000	No	ns	>0.9999

L. ExlA-treated pre-challenge lung IL-17A:

Dunn's multiple comparisons test	Mean rank diff.	Significant?	Summary	Adjusted P Value
PBS vs. E10 2.5 ug dmLT	-48.00	Yes	****	<0.0001
PBS vs. E1 2.5 ug dmLT	-41.20	Yes	***	0.0005
PBS vs. E10 L-PaF1 BECC/ME	-34.10	Yes	**	0.0076
PBS vs. E1 L-PaF1 BECC/ME	-36.90	Yes	**	0.0027
PBS vs. L-PaF1 BECC/ME	-27.00	No	ns	0.0770
PBS vs. E10	-11.60	No	ns	>0.9999
PBS vs. E1	-9.000	No	ns	>0.9999
PBS vs. L-PaF1	-20.80	No	ns	0.4009
PBS vs. PaF1 2.5 ug dmLT	-22.00	No	ns	0.2991
PBS vs. PaF1	-2.400	No	ns	>0.9999

Supplemental Table 2. Statistical analyses of MSD assay from pre-challenge young mice. See text for details.

Supplemental Table 3: MSD:

A. Post-challenge (CEC124) untreated lung IL-2:

Dunn's multiple comparisons test	Mean rank diff.	Significant?	Summary	Adjusted P Value
PBS vs. ExlA 10 + 2.5 ug dmLT	19.40	No	ns	0.5554
PBS vs. ExlA 1 + 2.5 ug dmLT	15.00	No	ns	>0.9999
PBS vs. ExlA 10 + L-PaF 1 BECC/ME	29.00	Yes	*	0.0421
PBS vs. ExlA 1 + L-PaF 1 BECC/ME	19.00	No	ns	0.6077
PBS vs. L-PaF 1 BECC/ME	6.400	No	ns	>0.9999
PBS vs. ExlA 10	-6.800	No	ns	>0.9999
PBS vs. ExlA 1	6.200	No	ns	>0.9999
PBS vs. L-PaF 1	17.20	No	ns	0.8960
PBS vs. PaF 1 + 2.5 ug dmLT	30.20	Yes	*	0.0288

PBS vs. PaF 1	-1.400	No	ns	>0.9999

B. Post-challenge (CEC124) untreated lung IFN-γ:

Dunn's multiple comparisons test	Mean rank diff.	Significant?	Summary	Adjusted P Value
PBS vs. ExlA 10 + 2.5 ug dmLT	-30.00	Yes	*	0.0307
PBS vs. ExlA 1 + 2.5 ug dmLT	-33.40	Yes	**	0.0098
PBS vs. ExlA 10 + L-PaF 1 BECC/ME	-17.60	No	ns	0.8239
PBS vs. ExlA 1 + L-PaF 1 BECC/ME	-18.60	No	ns	0.6640
PBS vs. L-PaF 1 BECC/ME	-27.60	No	ns	0.0645
PBS vs. ExlA 10	-16.60	No	ns	>0.9999
PBS vs. ExlA 1	-4.800	No	ns	>0.9999
PBS vs. L-PaF 1	-24.20	No	ns	0.1692
PBS vs. PaF 1 + 2.5 ug dmLT	-24.20	No	ns	0.1692
PBS vs. PaF 1	-1.000	No	ns	>0.9999

C. Post-challenge (CEC124) untreated lung IL-6:

Dunn's multiple comparisons test	Mean rank diff.	Significant?	Summary	Adjusted P Value
PBS vs. ExlA 10 + 2.5 ug dmLT	-5.200	No	ns	>0.9999
PBS vs. ExlA 1 + 2.5 ug dmLT	0.8000	No	ns	>0.9999
PBS vs. ExlA 10 + L-PaF 1 BECC/ME	-6.800	No	ns	>0.9999
PBS vs. ExlA 1 + L-PaF 1 BECC/ME	-7.600	No	ns	>0.9999
PBS vs. L-PaF 1 BECC/ME	0.000	No	ns	>0.9999
PBS vs. ExlA 10	-12.80	No	ns	>0.9999
PBS vs. ExlA 1	8.200	No	ns	>0.9999
PBS vs. L-PaF 1	-15.20	No	ns	>0.9999
PBS vs. PaF 1 + 2.5 ug dmLT	-11.00	No	ns	>0.9999
PBS vs. PaF 1	-3.200	No	ns	>0.9999

D. Post-challenge (CEC124) untreated lung TNF-α:

Dunn's multiple comparisons test	Mean rank diff.	Significant?	Summary	Adjusted P Value
PBS vs. ExlA 10 + 2.5 ug dmLT	-21.80	No	ns	0.3144
PBS vs. ExlA 1 + 2.5 ug dmLT	-14.60	No	ns	>0.9999
PBS vs. ExlA 10 + L-PaF 1 BECC/ME	0.4000	No	ns	>0.9999
PBS vs. ExlA 1 + L-PaF 1 BECC/ME	-5.200	No	ns	>0.9999
PBS vs. L-PaF 1 BECC/ME	-8.800	No	ns	>0.9999
PBS vs. ExlA 10	-7.400	No	ns	>0.9999
PBS vs. ExlA 1	0.8000	No	ns	>0.9999
PBS vs. L-PaF 1	-17.20	No	ns	0.8960
PBS vs. PaF 1 + 2.5 ug dmLT	-4.400	No	ns	>0.9999
PBS vs. PaF 1	-9.800	No	ns	>0.9999

E. Post-challenge (CEC124) untreated lung IL-17A:

Dunn's multiple comparisons test	Mean rank diff.	Significant?	Summary	Adjusted P Value
PBS vs. ExlA 10 + 2.5 ug dmLT	-32.80	Yes	*	0.0121
PBS vs. ExlA 1 + 2.5 ug dmLT	-17.60	No	ns	0.8239
PBS vs. ExlA 10 + L-PaF 1 BECC/ME	-23.80	No	ns	0.1883
PBS vs. ExlA 1 + L-PaF 1 BECC/ME	-16.80	No	ns	0.9731
PBS vs. L-PaF 1 BECC/ME	-15.60	No	ns	>0.9999
PBS vs. ExlA 10	-24.80	No	ns	0.1438
PBS vs. ExlA 1	-4.600	No	ns	>0.9999
PBS vs. L-PaF 1	-16.60	No	ns	>0.9999
PBS vs. PaF 1 + 2.5 ug dmLT	-16.20	No	ns	>0.9999
PBS vs. PaF 1	-9.400	No	ns	>0.9999

F. Post-challenge (mPa08-31) untreated lung IL-2:

Dunn's multiple comparisons test	Mean rank diff.	Significant?	Summary	Adjusted P Value
PBS vs. E10 2.5 dmLT	27.00	No	ns	0.0771
PBS vs. E1 2.5 dmLT	13.20	No	ns	>0.9999
PBS vs. E10 L-PaF1 BECC/ME	2.800	No	ns	>0.9999
PBS vs. E1 L-PaF1 BECC/ME	-5.000	No	ns	>0.9999
PBS vs. L-PaF1 BECC/ME	23.80	No	ns	0.1883
PBS vs. E10	-7.800	No	ns	>0.9999
PBS vs. E1	-1.200	No	ns	>0.9999
PBS vs. L-PaF1	12.00	No	ns	>0.9999
PBS vs. PaF1 2.5 dmLT	3.400	No	ns	>0.9999
PBS vs. PaF1	2.200	No	ns	>0.9999

G. Post-challenge (mPa08-31) untreated lung IFN-γ:

Dunn's multiple comparisons test	Mean rank diff.	Significant?	Summary	Adjusted P Value
PBS vs. E10 2.5 dmLT	-3.600	No	ns	>0.9999
PBS vs. E1 2.5 dmLT	-14.80	No	ns	>0.9999
PBS vs. E10 L-PaF1 BECC/ME	-19.60	No	ns	0.5307
PBS vs. E1 L-PaF1 BECC/ME	-3.400	No	ns	>0.9999
PBS vs. L-PaF1 BECC/ME	-9.600	No	ns	>0.9999
PBS vs. E10	-13.40	No	ns	>0.9999
PBS vs. E1	10.20	No	ns	>0.9999
PBS vs. L-PaF1	2.800	No	ns	>0.9999
PBS vs. PaF1 2.5 dmLT	-14.80	No	ns	>0.9999
PBS vs. PaF1	-8.600	No	ns	>0.9999

H. Post-challenge (mPa08-31) untreated lung IL-6:

Dunn's multiple comparisons test	Mean rank diff.	Significant?	Summary	Adjusted P Value
PBS vs. E10 2.5 dmLT	7.800	No	ns	>0.9999

PBS vs. E1 2.5 dmLT	10.20	No	ns	>0.9999
PBS vs. E10 L-PaF1 BECC/ME	-9.000	No	ns	>0.9999
PBS vs. E1 L-PaF1 BECC/ME	19.80	No	ns	0.5069
PBS vs. L-PaF1 BECC/ME	4.000	No	ns	>0.9999
PBS vs. E10	-3.600	No	ns	>0.9999
PBS vs. E1	-23.60	No	ns	0.1985
PBS vs. L-PaF1	-14.60	No	ns	>0.9999
PBS vs. PaF1 2.5 dmLT	-6.800	No	ns	>0.9999
PBS vs. PaF1	-8.400	No	ns	>0.9999

I. Post-challenge (mPa08-31) untreated lung TNF-α:

Dunn's multiple comparisons test	Mean rank diff.	Significant?	Summary	Adjusted P Value
PBS vs. E10 2.5 dmLT	-28.20	No	ns	0.0538
PBS vs. E1 2.5 dmLT	-26.20	No	ns	0.0972
PBS vs. E10 L-PaF1 BECC/ME	-33.60	Yes	**	0.0091
PBS vs. E1 L-PaF1 BECC/ME	8.200	No	ns	>0.9999
PBS vs. L-PaF1 BECC/ME	-25.40	No	ns	0.1218
PBS vs. E10	-7.000	No	ns	>0.9999
PBS vs. E1	-6.800	No	ns	>0.9999
PBS vs. L-PaF1	-18.40	No	ns	0.6938
PBS vs. PaF1 2.5 dmLT	-12.40	No	ns	>0.9999
PBS vs. PaF1	-13.00	No	ns	>0.9999

J. Post-challenge (mPa08-31) untreated lung IL-17A:

Dunn's multiple comparisons test	Mean rank diff.	Significant?	Summary	Adjusted P Value
PBS vs. E10 2.5 dmLT	9.200	No	ns	>0.9999
PBS vs. E1 2.5 dmLT	7.600	No	ns	>0.9999
PBS vs. E10 L-PaF1 BECC/ME	-3.800	No	ns	>0.9999
PBS vs. E1 L-PaF1 BECC/ME	9.800	No	ns	>0.9999
PBS vs. L-PaF1 BECC/ME	-0.2000	No	ns	>0.9999
PBS vs. E10	4.600	No	ns	>0.9999
PBS vs. E1	21.40	No	ns	0.3468
PBS vs. L-PaF1	31.40	Yes	*	0.0194
PBS vs. PaF1 2.5 dmLT	31.00	Yes	*	0.0222
PBS vs. PaF1	14.40	No	ns	>0.9999

Supplemental Table 3. Statistical analyses of MSD assay from post-challenge young mice. See text for details.

Supplemental Table 4.

Pre-challenge untreated lung IL-2:

Dunn's multiple comparisons test	Mean rank diff.	Significant?	Summary	Adjusted P Value
PBS vs. ExlA 10 + 2.5 ug dmLT	-21.00	No	ns	0.3821

PBS vs. ExlA 1 + 2.5 ug dmLT	-34.00	Yes	**	0.0079
PBS vs. ExlA 10 + L-PaF 1 BECC/ME	-17.60	No	ns	0.8239
PBS vs. ExlA 1 + L-PaF 1 BECC/ME	-38.80	Yes	**	0.0013
PBS vs. L-PaF 1 BECC/ME	-37.40	Yes	**	0.0022
PBS vs. ExlA 10	-3.200	No	ns	>0.9999
PBS vs. ExlA 1	-5.800	No	ns	>0.9999
PBS vs. L-PaF 1	-20.20	No	ns	0.4620
PBS vs. PaF 1 + 2.5 ug dmLT	-38.00	Yes	**	0.0018
PBS vs. PaF 1	0.4000	No	ns	>0.9999

Pre-challenge untreated lung IFN-γ:

Dunn's multiple comparisons test	Mean rank diff.	Significant?	Summary	Adjusted P Value
PBS vs. ExlA 10 + 2.5 ug dmLT	-30.60	Yes	*	0.0253
PBS vs. ExlA 1 + 2.5 ug dmLT	-39.40	Yes	**	0.0010
PBS vs. ExlA 10 + L-PaF 1 BECC/ME	-18.00	No	ns	0.7566
PBS vs. ExlA 1 + L-PaF 1 BECC/ME	-25.00	No	ns	0.1361
PBS vs. L-PaF 1 BECC/ME	-19.20	No	ns	0.5811
PBS vs. ExlA 10	2.200	No	ns	>0.9999
PBS vs. ExlA 1	-4.600	No	ns	>0.9999
PBS vs. L-PaF 1	-17.00	No	ns	0.9339
PBS vs. PaF 1 + 2.5 ug dmLT	-27.80	No	ns	0.0608
PBS vs. PaF 1	1.200	No	ns	>0.9999

Pre-challenge untreated lung IL-6:

Dunn's multiple comparisons test	Mean rank diff.	Significant?	Summary	Adjusted P Value
PBS vs. ExlA 10 + 2.5 ug dmLT	-20.80	No	ns	0.4009
PBS vs. ExlA 1 + 2.5 ug dmLT	-22.00	No	ns	0.2991
PBS vs. ExlA 10 + L-PaF 1 BECC/ME	-19.20	No	ns	0.5811
PBS vs. ExlA 1 + L-PaF 1 BECC/ME	-20.80	No	ns	0.4009
PBS vs. L-PaF 1 BECC/ME	-1.000	No	ns	>0.9999
PBS vs. ExlA 10	6.000	No	ns	>0.9999
PBS vs. ExlA 1	-6.800	No	ns	>0.9999
PBS vs. L-PaF 1	-18.80	No	ns	0.6354
PBS vs. PaF 1 + 2.5 ug dmLT	-14.60	No	ns	>0.9999
PBS vs. PaF 1	1.400	No	ns	>0.9999

Pre-challenge untreated lung TNF-α:

Dunn's multiple comparisons test	Mean rank diff.	Significant?	Summary	Adjusted P Value
PBS vs. ExlA 10 + 2.5 ug dmLT	-1.800	No	ns	>0.9999

PBS vs. ExlA 1 + 2.5 ug dmLT	-7.400	No	ns	>0.9999
PBS vs. ExlA 10 + L-PaF 1 BECC/ME	-5.600	No	ns	>0.9999
PBS vs. ExlA 1 + L-PaF 1 BECC/ME	-0.4000	No	ns	>0.9999
PBS vs. L-PaF 1 BECC/ME	5.400	No	ns	>0.9999
PBS vs. ExlA 10	12.20	No	ns	>0.9999
PBS vs. ExlA 1	10.40	No	ns	>0.9999
PBS vs. L-PaF 1	3.600	No	ns	>0.9999
PBS vs. PaF 1 + 2.5 ug dmLT	0.8000	No	ns	>0.9999
PBS vs. PaF 1	7.000	No	ns	>0.9999

Pre-challenge untreated lung IL-17A:

Dunn's multiple comparisons test	Mean rank diff.	Significant?	Summary	Adjusted P Value
PBS vs. ExlA 10 + 2.5 ug dmLT	-37.80	Yes	**	0.0019
PBS vs. ExlA 1 + 2.5 ug dmLT	-44.00	Yes	***	0.0001
PBS vs. ExlA 10 + L-PaF 1 BECC/ME	-21.00	No	ns	0.3821
PBS vs. ExlA 1 + L-PaF 1 BECC/ME	-33.20	Yes	*	0.0105
PBS vs. L-PaF 1 BECC/ME	-35.80	Yes	**	0.0041
PBS vs. ExlA 10	-6.000	No	ns	>0.9999
PBS vs. ExlA 1	-9.000	No	ns	>0.9999
PBS vs. L-PaF 1	-22.00	No	ns	0.2991
PBS vs. PaF 1 + 2.5 ug dmLT	-35.80	Yes	**	0.0041
PBS vs. PaF 1	-6.200	No	ns	>0.9999

Supplemental Table 4. Statistical analyses of MSD from pre-challenge elderly mice. See text for details.

Supplemental Table 5: Unique genes (up, and downregulated), and their probable functions (REF):

Only present in CEC124 infected mouse lung	Probable function of the gene (or the gene product)	Fold-change	Only present in mPa08-31 mouse lung	Probable function of the gene (or the gene product)	Fold-change
Pus3	Enables pseudouridine synthase activity, involved in RNA modification.	+10.29042	Saa3	Enables TLR4 binding, and chemoattractant activity.	+10.38196
Pcdhga10	Involved in cell adhesion.	+10.44279	1810049J17Rik	Has not yet been conclusively described.	+10.29112
Ccl20	Enables chemokine activity.	+10.45707	H4c6	Structural constituent of chromatic, enables protein	+10.26461

				domain specific binding.	
Saa1	Enables G protein-coupled receptor binding, acts upstream of r within acute-phase response.	+10.43336	Wdr38	Acts upstream of hematopoietic progenitor cell differentiation.	+10.019
Fbp1	Involved in carbohydrate metabolism.	+11.39671	Camp	Enables LPS binding, involved in antibacterial humoral response.	+10.01813
Psd	Enables guanyl-nucleotide exchange factor activity.	-5.122208	Il1f6	Involved in cellular response to LPS.	+10.10977
mt-Nd6	Enables NADH dehydrogenase activity, involved in aerobic respiration.	-6.34035	Esm1	Enables hepatocyte growth factor receptor binding, and integrin binding.	-5.4251
Cyp26b1	Enables metal binding.	-5.16787	Hpcal4	Enables calcium channel regulator activity.	-6.04801
Mt-Nd4l	Enables NADH dehydrogenase activity, involved in aerobic respiration.	-5.21149	Rps26-ps1	No defined function.	-5.14012
Mt-Tc	Involved in translational elongation.	-6.58226			
Mt-Te	Involved in translational elongation.	-6.70938			
Cyp8b1	Enables metal binding.	-6.3507			
Mt-Tl2	Involved in translational elongation.	-8.17332			
Igkv3-7	Immunoglobulin production.	-5.49722			
Igkv6-20	Immunoglobulin production.	-8.59763			
H1f10	Involved in chromatin condensation.	-5.05333			
Rapgef4os3	Enables molecular function.	-6.27278			
Rasl10a	Enables G protein activity.	-5.2259			

Asic1	Involved in ion-channel activity.	-5.09646			
4930516B21Rik	Enables ATP binding.	-5.97029			
Igkv6-32	Immunoglobulin production.	-6.71227			
Kcnh7	Enables potassium channel activity.	-7.35379			
C130083M11Rik	Enables molecular function.	-5.86085			
Gkn3	Enables molecular function.	-7.23745			
Mt-Ts2	Involved in translational elongation.	-5.94569			
5031425F14Rik	Enables molecular function.	-5.92863			
Rps11-ps2	No defined function.	-7.05734			
4933413J09Rik	Enables molecular function.	-5.03689			
4930565N06Rik	Enables molecular function.	-6.53287			
Igkv4-72	Immunoglobulin production.	-5.60615			

Supplemental Table 5. Unique gene profile of elderly mice lung post-challenge. Elderly mice were challenged with either CEC124, or mPa08-31, lungs removed, and mRNA sequenced. Genes were filtered based on the exclusion of the term 'GM.' Genes were further filtered based on their up/downregulation fold-change. Genes with more than equal 10-fold upregulation, and less than equal 5-fold downregulation were taken into consideration. Unique genes have been tabulated with their probable functions.
